# Movement-Based Low Back Pain Subgroups Using Motion Tape Strain Data with Biomechanical and Causal Feature Engineering

**DOI:** 10.3390/s26123800

**Published:** 2026-06-15

**Authors:** Aarti Lalwani, Sara P. Gombatto, Yasmin Velazquez, Elijah Wyckoff, Pratham Yashwante, Kevin Patrick, Kenneth J. Loh, Rose Yu, Emilia Farcas

**Affiliations:** 1 Computer Science and Engineering, University of California San Diego, La Jolla, CA 92093, USA; adlalwani@ucsd.edu (A.L.); pyashwante@ucsd.edu (P.Y.); roseyu@ucsd.edu (R.Y.); 2 School of Physical Therapy, San Diego State University, San Diego, CA 92182, USA; sgombatto@sdsu.edu; 3 School of Exercise and Nutritional Sciences, San Diego State University, San Diego, CA 92182, USA; yvelazquez3@sdsu.edu; 4 Active, Responsive, Multifunctional, and Ordered-Materials Research (ARMOR) Laboratory, Department of Structural Engineering, University of California San Diego, La Jolla, CA 92093, USA; ewyckoff@ucsd.edu (E.W.); kenloh@ucsd.edu (K.J.L.); 5 Qualcomm Institute, University of California San Diego, La Jolla, CA 92093, USA; kpatrick@ucsd.edu; 6 School of Public Health, University of California San Diego, La Jolla, CA 92093, USA

**Keywords:** machine learning, motion sensing, wearable sensors, time-series analysis, low back pain, movement-based subgroups, unsupervised learning, causal discovery

## Abstract

Low back pain (LBP) is a major global health problem and can result in a variety of movement impairments. Advances in smart technology have enabled the collection of novel streams of movement data, and machine learning (ML) methods have been increasingly used for data analysis. However, many existing technologies remain expensive and unsuitable for widespread clinical use, and ML approaches have largely focused on distinguishing people with LBP from healthy controls rather than identifying meaningful subgroups within the LBP population. Motion Tape (MT) is a recently developed wearable strain sensor that translates skin deformation from underlying movement and muscle engagement into electrical signals. In this exploratory study involving 10 participants with LBP, we demonstrate that MT data from six sensors applied on the lower back capture rich movement information capable of characterizing movement patterns among participants with LBP. We propose a feature engineering approach based on biomechanical features as well as time-series causal discovery applied to multivariate sensor time-series data to extract directed inter-segment coordination patterns. We further develop an exploratory subgroup discovery pipeline by aggregating clustering coassociation information across diverse movement tasks. Our causal coordination features show promising discriminative information across several movement types, capturing aspects of motor control not reflected in amplitude-based or embedding-based features alone, such as asymmetries and movement restrictions. Preliminary ensemble clustering analysis indicates three potential LBP subgroups distinguished by biomechanical and inter-segment coordination patterns, which may reflect varied strategies under different movement demands. We investigate the differences in clinical characteristics among these LBP subgroups. We show that time-series foundation models are not well suited for LBP subgrouping due to their uninterpretability, which is improved in our feature engineering pipeline. This framework could reveal additional subgroups with larger cohorts and may generalize to other sensor modalities.

## 1. Introduction

Low back pain (LBP) is one of the leading causes of disability globally and affects individuals across a wide range of demographic groups [1]. LBP substantially reduces quality of life, limits the ability to perform basic daily activities, and is associated with adverse mental health, poor sleep health, and increased medication use. Younger populations, particularly those with poor posture during prolonged sitting when using electronic devices, are experiencing LBP at increasingly earlier ages [2]. Middle-aged individuals in both physically demanding and highly sedentary occupations are also heavily affected, and age-related musculoskeletal changes contribute to LBP in older adults [2]. In the United States alone, the economic burden of LBP is estimated to exceed USD 100 billion dollars per year [2].

LBP is associated with altered movement patterns, including stiffness, compromised speed and variability of movement, reduced range of motion in multiple planes, as well as altered trunk muscle activation [3,4,5,6]. Movement assessment is a key component of clinical evaluation by physical therapists to determine the severity of LBP, identify specific movements that are associated with LBP, and monitor the progress of physical therapy. Physical therapy interventions typically focus on enhancing muscle activation, strength, stability, and mobility to reduce pain and the risk of disability [7,8,9]. Although physical therapy is effective for treating LBP, challenges include personalizing exercises to each patient’s needs and assessing movement patterns at home.

With advances in sensing technologies, machine learning (ML) methods are increasingly being applied to analyze these sensor streams for LBP-related data. However, much of the literature prioritizes distinguishing LBP from control populations with limited studies focused on identifying subgroups within the LBP population [10].

### 1.1. Problem Setting and Challenges

This paper explores whether data from remote sensors can be analyzed with ML tools to identify movement-based LBP subgroups. To acomplish this, sensor data for movement analysis must capture the multi-segmental nature of the lumbar spine across all three planes of motion. Additionally, sensors should consistently capture a linear and reversible response over a wide range of movement to accurately capture both small and large movements. Traditional systems such as surface electromyography (sEMG) and inertial measurement unit sensors (IMUs) provide accurate measurements but are often expensive and bulky. Flexible wearable sensors with piezoresistive materials have the ability to conform to the body, making them well suited for monitoring complex, multi-segmental movements in daily activities.

Motion Tape (MT) is a wearable piezoresistive skin-strain sensor that has demonstrated potential for LB movement analysis. MT is fabricated by depositing graphene nanosheets or carbon nanotubes onto a commercial kinesiology tape (k-tape) substrate, enabling a flexible, comfortable, and cost-effective solution for capturing movement data [11,12,13]. The technology translates skin deformation resulting from joint motion and muscle engagement into electrical signals, and its direct application to the skin reduces motion artifacts. MT demonstrates high sensitivity, linearity, and consistency, and it enables spatial strain sensing across larger regions through a multi-sensor configuration [11,12,13]. As MTs are custom fabricated and are improved based on the feedback from each study, large-scale data collection is not yet feasible.

### 1.2. Contributions

The goal of this paper is to identify preliminary movement-based LBP subgroups using multivariate time-series data collected with a network of MTs. We employ unsupervised learning, without any ground truth labels about LBP subgroups, as data streams from MT sensors might identify new or different LBP subgroups than clinicians or previous motion capture studies. This is because prior research did not benefit from the finer grained data on movement patterns available from MTs.

In this exploratory study involving 10 participants with LBP, we provide preliminary evidence that data from six MTs placed on the lower back capture rich information about underlying biomechanical and coordination-related qualities that can be used to characterize movement patterns in individuals with LBP. Enabled by MT grid-based sensor placement, this paper is, to our knowledge, among the first to (a) leverage low back sensor data to characterize spatiotemporal coordination patterns during movement using time-series causal discovery methods; (b) integrate these with biomechanical features; and (c) explore cross-movement clustering to identify movement-based LBP subgroups.

The main contributions are as follows. (i) We propose a new feature engineering approach for LBP subgrouping using standard biomechanical signal features as well as applying PCMCI+ (Peter and Clark Momentary Conditional Independence) [14] to multivariate sensor time-series data to extract inter-segment coordination patterns. (ii) We present a novel cross-movement subgroup discovery pipeline that applies per-movement clustering (k-means [15] and agglomerative clustering [16]) to construct cross-movement coassociation matrices, followed by ensemble clustering (hierarchical [17] and spectral clustering [18]) to identify subgroups, while mitigating small-sample challenges through cluster stability assessment. (iii) We identify preliminary evidence of three LBP subgroups that are distinguished by movement characteristics and coordination strategies: a majority subgroup exhibiting moderate trunk movement and two smaller distinctive subgroups, characterized by lateral asymmetry and greater overall displacement.

## 2. Related Work

Improvements in sensing technologies have expanded the scope of movement-based LBP research and have facilitated the increasing application of ML for LBP analysis [19,20,21,22,23,24]. A systematic review states that much of the literature focuses on distinguishing LBP from control populations rather than on subgroup identification within LBP, highlighting the need to further explore clinically meaningful LBP subgroups [10]. Existing studies face several limitations, such as a reliance on resource-intensive data collection systems and focus on a limited set of movements. The majority of studies rely on IMU sensors or marker-based motion capture systems with markerless motion capture remaining underutilized despite its clinical potential [10]. While marker-based motion capture provides high spatial accuracy, it requires specialized laboratory environments, trained operators, and significant setup time, limiting its accessibility for routine clinical assessment or large-scale studies [25,26]. Movement-specific LBP subgroup studies often report limited or no differentiation of kinematic data between subgroups [27,28,29]. Furthermore, subgroup definitions vary widely and include factors such as pain characteristics, treatment [30], risk profiles [31], disease progression [32] and movement impairments [3,33].

Traditional systems such as IMUs and sEMG provide accurate measurements but serve different purposes [34,35,36,37,38]. IMUs capture global lumbar kinematics but have limited ability to detect inter-segmental spinal motion or local tissue deformation [31,39,40,41]. Surface EMG captures muscle activation patterns [42] but is sensitive to electrode placement and signal cross-talk and does not capture the resultant movement. MT measures local mechanical response to skin deformation as a result of underlying segmental spine motion and muscle engagement. Its piezoresistive design and direct skin adhesion offer advantages for comfort, conformability, and artifact reduction in free-living conditions due to direct skin adhesion.

MT is a wearable piezoresistive skin-strain sensor that exhibits high sensitivity, linearity, stability, and repeatability [11,12]. Loading experiments demonstrated consistent strain responses and stable sensor performance across rotation, forward flexion, and lateral bending tasks [12]. MT has been further validated, demonstrating high correlation with motion capture data for lower back movements and capability of capturing muscle engagement during movement in individuals without LBP [13,43]. Additionally, spatial strain sensing can be achieved through strip or mesh sensor configurations, enabling the mapping of muscle activity across larger regions and demonstrating potential for tracking muscle group engagement during diverse tasks [11].

Prior research has leveraged MT data for deep learning-based movement classification, including its use as input to convolutional autoencoders for detecting incorrect movements in marksmanship and golf tasks [44,45]. In the context of LBP, conditional generative models applied to MT data have also shown effectiveness in classifying movements such as extension, flexion, lateral bending, and rotation [46].

## 3. Methods

### 3.1. Human Movement Study Design

A total of 10 participants with chronic LBP were included in this paper. Inclusion criteria were defined as the following: age between 18 and 65, presence of chronic LBP (defined by the NIH Research Task Force [47] as pain persisting for at least three months and occurring on at least half of the days over the past six months), ability to follow movement instructions in English or Spanish, and capacity to perform simple trunk movements and functional activities like walking. The cohort had a mean age of 42.0±16.7 years, height of 172.1±7.3 cm, weight of 80.7±23.2 kg, and waist–hip ratio of 0.83±0.1. The participants were 60% female and 40% male and included individuals from diverse racial backgrounds. Five participants reported LBP duration greater than 5 years, four between 1 and 5 years, and one less than 1 year.

**Figure 1:**
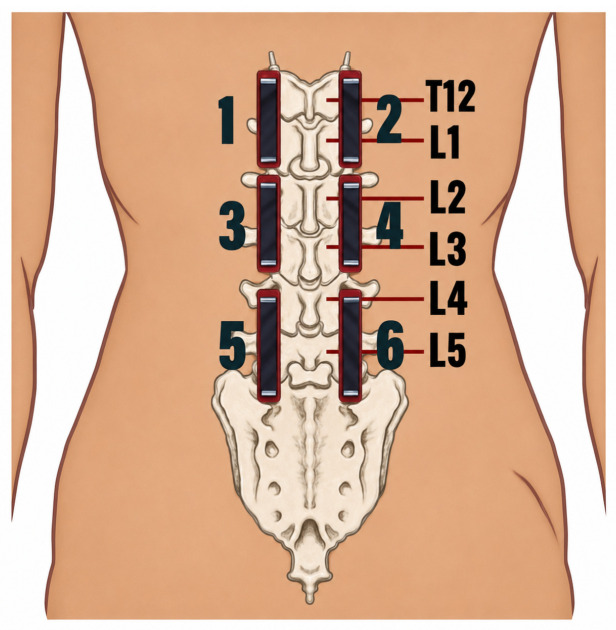
Placement of the 6 MTs. First, the middle MTs 3 and 4 were positioned across the L2 to L3 and L3 to L4 junctions with the bottom edges placed just above L4. Then, the top MTs 1 and 2 were placed above the middle sensors across T12 to L1 and L1 to L2, and finally the bottom MTs 5 and 6 were placed below the middle sensors across L4 to L5 and L5 to S1.

An experienced physical therapist advised MT placements according to key lumbar spine and pelvic landmarks. Six MTs were placed in a 3 row by 2 column grid on each participant’s thoracolumbar and lumbosacral regions spanning from T12 to S1, with sensors placed on either side of the spine, as depicted in Figure 1. There was minimal separation vertically between MTs: the middle MTs were placed first and then the upper and lower MTs were placed directly adjacent to the middle MTs to allow minimal separation without overlap. The same study investigator placed MTs for all participants and was trained by the physical therapist investigator.

Participants were instructed to perform a series of movements which included isolated clinical movement tests and more complex functional movements in a variety of positions and which captured different movement directions, as shown in Table 1. Each movement was repeated 3 times except for stair climbing, walking, and driving. MT data were recorded approx. every 17 ms. Participants moved at a self-selected speed; the recording duration for each movement averaged 31.2 s across participants. Participants also provided self-reported ratings of pain increase during each movement trial.

### 3.2. MT Fabrication and Data Processing

MTs were fabricated as previously described in [12]. Rectangular k-tape (Rock Tape, Durham, NC, USA) of 40 × 60 mm^2^ was masked to design the sensing area of 7.5 × 40 mm^2^. This paper used multi-walled carbon nanotubes (MWCNTs) instead of the graphene nanosheets from earlier studies, as MWCNT-MTs were shown to exceed GNS-MTs in terms of consistency of signal stability [12]. First, the k-tape substrate was treated with ethanol. Then, a 2% MWCNT aqueous dispersion was drop-cast and brushed onto the sensing area in four successive layers, each layer containing 0.25 mL of MWCNT dispersion. The sensing element dried over 12 h. This formed a percolated network of electrically conductive pathways into the k-tape textile fibers. Finally, conductive silver paste (Conductor 3 Silver Ink from Voltera, Kitchener, ON, Canada) was applied to each end of the sensing element to form electrodes, and multistrand wires (from Digi-Key, Thief River Falls, MN, USA) were soldered to the electrodes. The data acquisition system consisted of an ESP32 microcontroller and an analog-to-digital converter (Analog Devices, Wilmington, MA, USA) and transmitted resistance measurements wirelessly using Bluetooth Low Energy (BLE).

The performance of MT sensors was previously evaluated [12]. Sensor consistency in sample preparation was evaluated with controlled electromechanical cyclic loading tests for four samples, which demonstrated that MWCNT-MT exhibited low standard deviations (0.074) across strain levels of 1–8%. Strain rate sensitivity and signal stability under repeated loading were evaluated with cyclic strain tests at loading rates of 10–50% of max strain/second, which showed that the strain rate has a minimal effect on peak resistance. Signal stability was validated through 100-cycle tests. Further human movement analysis showed that sensors maintained signal stability during all movements even at higher strains.

Skin strain is quantified from resistance changes measured by the MT sensors. Sensor resistance data were normalized using $\left(\frac{R_{t}}{R_{\mathrm{initial}}}\right)-1$, with $R_{\mathrm{initial}}$ defined as the pre-trial baseline measurement, when the participant is at rest and the sensor is unstretched, and $R_{t}$ defined as the electrical resistance measured at time *t*. This converts raw resistance values into a ratio representing proportional change from a baseline state, so the data are reflective of skin strain during the movement trial. Normalizing to baseline resistance accounts for sensor-to-sensor variability in resting resistance due to manufacturing differences. Furthermore, baseline resistance was recorded prior to each movement type to eliminate effects from one movement to another due to stretching of the sensing element, sensor adhesion, or sensor drift.

**Figure 2:**
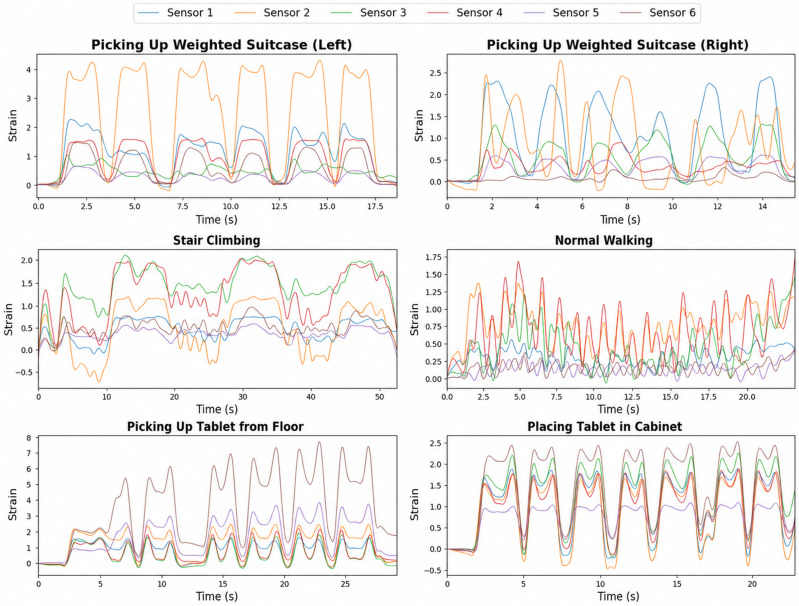
MT signals for one subject for six movements.

Data were sampled at a frequency of 60 Hz. MT resistance data were read into MATLAB R2023a, converted to decimal, and normalized. The resulting time series were filtered using a Hampel algorithm to discard outliers. Any later data processing for specific feature engineering is described in Section 3.4 and Section 3.5. Figure 2 shows an example of data streams from all 6 sensors for one participant performing 6 functional movements.

### 3.3. Overview of LBP Subgrouping Approach

**Figure 3:**
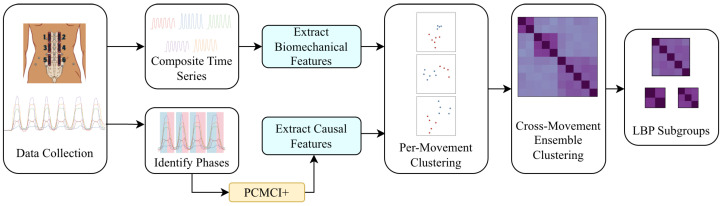
LBP subgrouping framework. Six MTs measure lumbar strain during movement trials. Biomechanical features are extracted from composite series, and causal features are derived by applying PCMCI+ to phase-segmented signals. Combined features are used for per-movement clustering; then, cross-movement ensemble clustering via coassociation matrices identifies LBP subgroups.

Our approach is described in Figure 3. We first capture strain signals from six MTs during movement trials. From the six sensor signals, we construct five composite time series representing spatial aggregations (means of left, right, top, and bottom MTs and overall magnitude), which are used to extract biomechanical features. Furthermore, the original sensor signals are used to identify phases by segmenting each movement repetition into a primary phase (baseline to peak strain) and a return phase (peak strain back to baseline), which are used to extract features with inter-sensor causal interpretations. Then, we combine the biomechanical and causal features, and we perform clustering separately per movement, since different movements require distinct spinal coordination patterns and may reveal different aspects of subgroup structure. These results can be represented as a coassociation matrix [48], which is then clustered as part of a cross-movement ensemble approach to form overall LBP subgroups with similar movement patterns across movements.

We adopted this two-step clustering approach rather than directly clustering all movements together because each movement type engages the spine differently, producing discriminative features in different subsets of the feature space. Clustering all movements together would risk allowing features from certain movement types to dominate, potentially obscuring subgroup structure from other movements. Initial per-movement clustering allows task-relevant features to drive the groupings for each movement independently. The subsequent coassociation step identifies subjects who are consistently grouped together across diverse movements, providing a more robust basis for finding shared coordination strategies than a single pooled clustering.

In the remainder of this section, we detail the biomechanical and causal feature engineering process, and we address data assumptions. We then formalize the subgroup discovery method. In Section 4, we present the results of our experiments, beginning with the weaknesses of time-series foundation models for LBP subgrouping as a motivating factor for our proposed pipeline. We then analyze clustering quality metrics and provide results of movement-specific and multiple-movement differentiating features, which we use to characterize subgroups. We also make observations about clinical characteristics and pain differences across subgroups. Finally, in Section 5 and Section 6, we discuss the limitations of this paper and overall conclusions.

### 3.4. Biomechanical Feature Engineering

**Figure 4:**
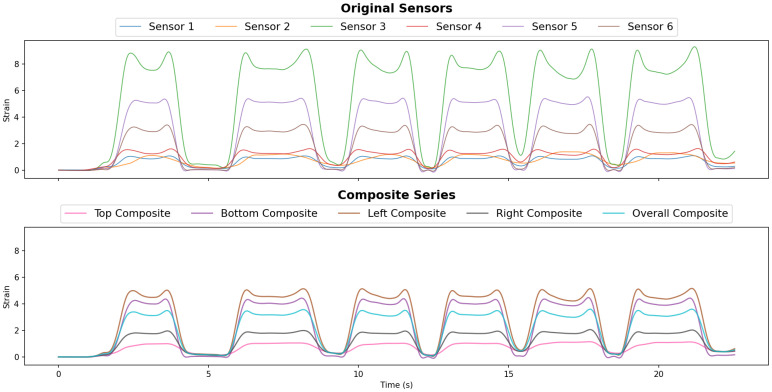
Original MT signals and composite series corresponding to one subject’s repetitions for placing tablet in cabinet.

From the six MT signals, we constructed five composite time-series signals that reflect clinically meaningful spatial aggregations: top mean (upper-row sensors 1 and 2), bottom mean (lower-row sensors 5 and 6), left mean (left-column sensors 1, 3, and 5), right mean (right-column sensors 2, 4, and 6), and overall magnitude (all six sensors). The overall magnitude was calculated as the Euclidean norm across the six sensor channels at each timestep, $m\left(t\right)=\sum_{i=1}^{6}x_{i}{\left(t\right)}^{2}$. Figure 4 shows an example of the original six sensor signals and the five composite series for one movement. These composite series capture patterns relevant to our movement set. The top and bottom composites reflect the vertical distribution of strain, which is relevant to forward bending depth and vertical trunk positioning during all movements. The left and right composites capture lateral asymmetries that may manifest during lateral bending, asymmetric gait patterns, or compensatory trunk shifts during forward bending or other functional movements. The overall composite provides a global measure of movement across all tasks. Notably, strain data can be positive (indicating sensor tension) or negative (indicating sensor compression).

Table 2 presents the biomechanical features we extracted from the composite time series with their exact formulations and interpretations. Briefly, from each of the five composite time series, we extracted three features to represent range, maximum rate of change, and mean near-peak duration. Additionally, we calculated jerk, as a measure of movement smoothness, from the overall composite. We also calculated the coefficient of variation for both peak amplitude and inter-peak interval (time between consecutive peaks) to capture the repetition variability within each trial. Furthermore, two features were computed to capture left–right and top–bottom asymmetry, which are each defined as the normalized difference between the mean values of the respective composite series [49,50]. In total, we constructed 20 biomechanical features per sample.

### 3.5. Causal Feature Engineering via PCMCI+ and Movement Selection

As noted in the Introduction, LBP can manifest with varied movement patterns. This can include guarded or stiff behavior, possibly indicated by overall reduced strain and dense inter-sensor relationships, as well as compensatory mechanisms, such as asymmetric strain patterns [4,6]. Beyond traditional biomechanical signal features, causal relationships between low back sensors may reveal how different spinal regions interact during movement, potentially capturing patterns that distinguish LBP subgroups. Therefore, we applied PCMCI+, a causal discovery algorithm for time series [14], to derive these directed coordination patterns between spinal regions, which we then use to characterize LBP subgroups.

#### 3.5.1. Overview of PCMCI+

PCMCI+ is designed to discover both lagged and contemporaneous relationships, handles autocorrelation, and accounts for time resolutions that might be coarser than true temporal relationships. These characteristics make the algorithm well suited for continuous time-series strain signals from the 6-sensor network of MTs measuring low back strain. PCMCI has two key stages: the first, based on the PC algorithm, identifies potential causal parents of each variable, and the second uses the Momentary Conditional Independence test to prune links. PCMCI+ modifies these stages by considering lagged and contemporaneous sets separately and optimizing the conditional independence tests. PCMCI+ has been used to analyze joint dynamics (knee, trunk, and ankle) during movement trials in osteoarthritis research [51], but to our knowledge, it has not been applied in LBP studies. The high sampling rate (60 Hz) and spatial configuration of the sensors provide dense spatiotemporal data well suited for applying this algorithm to study inter-segment coordination in the low back.

We adopt the graphical causal modeling framework [52], which, for causal inference from observational data, depends on standard assumptions. These are causal sufficiency (relevant common causes are observed), the causal Markov condition (a variable is independent of its non-descendants given its parents), and the faithfulness assumption (all conditional independence is due to the causal graph structure). While these assumptions are standard in causal discovery, they may only be approximately satisfied in real-world observational data and cannot be formally verified [53]. Nevertheless, we argue that they are reasonable in this context. Causal sufficiency is supported by the inclusion of all sensors across the region of interest and by accounting for participant-specific baseline resistance through normalization, thereby ensuring that baseline differences do not confound the relationships captured in the data [53]. The causal Markov condition is reasonable given that the measured signals capture the relevant strain dynamics of the system [54]. Similarly, the faithfulness assumption is plausible, as the observed strain time series are expected to reflect the underlying biomechanical relationships of interest [11,12,13,43,54]. In addition to the preceding standard assumptions, PCMCI+ operates under the fundamental assumption of causal stationarity (the causal relationships do not change over time). Stationarity can be explicitly tested [55], and we use this for movement set selection, which is described later in this section.

A common challenge for causal discovery in dynamic systems is that relationships between variables may shift depending on the background regime, which can be modeled as switching states [56]. While research has been conducted to automatically detect such regime changes in settings like climate [56], our movement data offer a natural segmentation based on domain knowledge: each repetition consists of a primary phase (baseline to peak strain) and a return phase (peak strain back to baseline). The functional demands differ between these two phases: the trunk is displacing during the primary phase and returning to (bending movements) or establishing a new baseline (gait movements) during the return phase. Therefore, the underlying causal structure is expected to differ between them, but remain approximately stationary within each phase, across repetitions of the same movement type.

The dataset included heterogeneous movement types such as flexion, lateral bending, extension, rotation, gait, and combinations of these movement types required for some functional activities. During each movement repetition, the participant begins from baseline, enters the primary phase, completes the movement, which is indicated by the signal reaching peak strain, enters the return phase, and then returns to baseline before repeating, which lasts until the end of the trial. During each phase, different trunk regions undergo sensor strain changes at different times. For example, upper sensors may measure increasing strain before lower sensors during forward bending. PCMCI+ infers these temporal relationships and represents them as a causal graph: a directed link from sensor i to sensor j indicates that strain changes at sensor i systematically precede strain changes at sensor j within that phase, while contemporaneous links indicate sensors whose strain changes occur simultaneously. The causal features derived from these graphs characterize the spatiotemporal coordination of trunk strain, which is represented by the sequence and synchrony with which different low back region sensors measure strain during each phase of the movement.

#### 3.5.2. Movement Selection

We selected a subset of the movements with the strongest potential for coordination analysis through PCMCI+ by testing the stationarity of the primary and return phases of all participants for each movement. No standard protocol exists for assessing within-phase causal stationarity in movement biomechanics data. We adopted a split-half approach inspired by stationarity assessment methods from functional connectivity analysis and regime-dependent causal discovery [56,57]. Each participant’s phase-specific segments were sorted chronologically based on trial timing into early (1st half of trial) and late (2nd half of trial) segments. Stationarity was assessed by building the full pairwise correlation matrix separately for early and late repetitions and then computing two measures of inter-sensor correlation stability between early and late repetitions: mean correlation shift and correlation profile similarity. Mean correlation shift tests whether correlation magnitudes are stable, and it was quantified as the average absolute change in pairwise sensor correlations. Correlation profile similarity tests if correlation structure is preserved, and it was quantified as the Pearson correlation of the upper-triangular pairwise correlation values from the early and late matrices.

Movements were considered suitable for PCMCI+ analysis when mean correlation shifts were low and correlation profile similarity was high, indicating that the dependency structure remained approximately stationary across repetitions. Thresholds for stationarity ratings were defined empirically and were informed by previous stationarity assessment approaches [56,57]. Phases with correlation shift <0.15 and correlation profile similarity >0.8 were rated as good, whereas those with shift <0.25 and similarity >0.6 were rated as adequate. We used a minimum threshold of 100 timesteps per phase to determine sample sufficiency. The thresholds were chosen to balance the trade-off between retaining sufficient data for causal discovery and ensuring approximate stability of the within-phase causal structure. We calculated an overall stationarity score, ranging from 0 to 1, as the percentage of good and adequately stationary phases multiplied by the percentage of sufficiently long phases.

**Figure 5:**
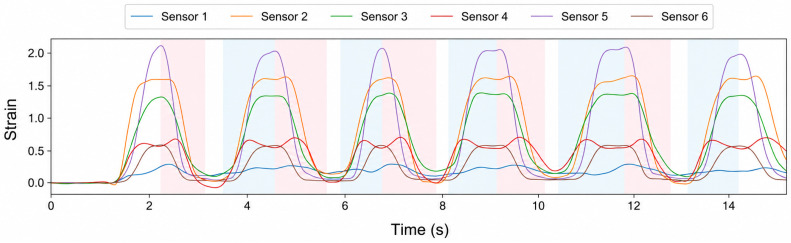
Example of primary (blue) and return (pink) phases refined by stationarity criteria for a subject’s repetitions for picking up weighted suitcase (right).

Figure 5 shows an example of the primary and return phases. Phase boundaries were refined using stationarity criteria. Initial primary and final return phases were excluded when their cross-correlation structures slightly differed from those of the intermediate phases of the trial. These differences may be attributable to multiple underlying biomechanical and behavioral factors. For example, in the initial primary phase, participants might be adjusting to the task, finding their range, and learning their required effort. The final return phase may involve an anticipation of stopping. We retained only the segments where within-phase causal stationarity was best supported.

Using the stationarity score depicted in Table 3, the top six movements were chosen as our movement set for analysis (they are further described in Table 4). The selected movements had a mean of 430–980 timesteps per phase, which is consistent with PCMCI+ and regime-based PCMCI studies that demonstrate good performance with overall time series or background phase lengths of a few hundred observations or more [14,56]. For movements performed bilaterally, it was required that both movements to the right and left sides meet the stationarity criteria. If only one side qualified, the pair was excluded to maintain a balanced movement set for the subgrouping coassociation matrix. For example, reverse driving left had a marginal stationarity score (0.52), but reverse driving right had a very low score (0.15), so both were excluded. Further excluded from consideration and not depicted in the table were movements with analogous variants already selected (picking up suitcase right/left was excluded, as picking up weighted suitcase right/left was included).

The selected movements capture diverse movement types in multiple planes of movement (see Table 4), including flexion and lateral bending. Thus, we consider this a sufficient set for our exploratory subgrouping study and use it as the focus of the following analysis. Notably, we did not include rotation movements, which demonstrated poor causal stationarity in primary and return phases. Intuitively, at the beginning of rotation, sensors on the stretched side show increased strain while sensors on the opposite side show compression with values near baseline, and this relationship reverses within a single phase as the rotation progresses. In contrast, during bending tasks, the sensors’ strains generally increase together in primary phases and decrease together in return phases. The change in strain during rotation violates the within-phase causal stationarity assumption required by PCMCI+. Further subdividing each phase to isolate stationary subsegments is challenging and it would substantially reduce each phase’s available data, limiting the reliability of causal discovery.

PCMCI+ supports mask arguments that restrict causal discovery to designated time segments where the causal structure is expected to be stationary. We leverage this when analyzing selected movements by constructing separate masks for primary and return phases and applying PCMCI+ separately for the two phases of each sample [53,55].

#### 3.5.3. Causal Feature Engineering

For each movement and participant, PCMCI+ outputs a causal graph, where the nodes are the six sensors. One matrix encodes the edge types as directed, undirected, ambiguous (where the algorithm could not resolve the causal orientation), or no link. It also outputs a value matrix encoding the partial correlation strength. Both are the same size, with the first two dimensions corresponding to the sensor indices, and the third dimension corresponding to the lag value (contemporaneous/simultaneous at t = 0, lagged at t > 0).

Table 5 details the features we constructed from the PCMCI+ results. For primary and return phases separately, we captured contemporaneous coupling topology, per-sensor net influence, and spatial asymmetries. The total number of contemporaneous links (directed, undirected, and ambiguous) was counted. These links were then classified by spatial orientation as the following: horizontal pairs (sensors in the same row), vertical pairs (sensors in the same column), and diagonal pairs (cross-column, cross-row). These were expressed as ratios of the total contemporaneous link count to characterize whether simultaneous inter-sensor coordination is predominantly lateral, vertical, or diagonal. Furthermore, for each sensor, the total number of outgoing directed edges (both lagged and contemporaneous) minus the total number of incoming directed edges provided a per-sensor net influence metric. This captures if each trunk segment mainly leads or follows other segments during each phase. The outgoing edge counts were aggregated by spatial grouping to compute left–right and top–bottom asymmetry. Diagonal–vertical balance was also calculated to represent the proportion of directed edges along diagonal versus vertical sensor pairs.

We also constructed features that represent how coordination reorganizes between primary and return phases. The absolute difference between phases was computed for each contemporaneous coupling ratio (horizontal, vertical, diagonal) and for the left–right and top–bottom spatial asymmetry indices. Larger values indicate that a participant’s coordination strategy changes substantially between movement phases. These features can be informative of cross-phase guarded movement and changes in coordination strategy when bending or moving in one direction versus the opposite direction when returning to baseline. This produced 13 per-phase features (for both primary and return) and 5 cross-phase comparison features, totalling 31 features for each movement and participant.

### 3.6. Per-Movement Clustering

We applied two clustering methods, as we could find no clearly established standard algorithm suited to this dataset. To discover clusters for each movement, we required clustering methods that were appropriate for our small sample size and accordingly chose k-means clustering [15] and agglomerative clustering [16]. We evaluate results for 2, 3, and 4 clusters using leave-one-subject-out (LOSO) cluster consistency [58,59] as a measure of stability and cluster predictability assessed via logistic regression as a complementary stability metric [60].

LOSO clustering consistency measures whether the cluster structure is robust to removing individual participants. For each participant, it is left out, the remaining participants are re-clustered, and the left-out participant is assigned to the nearest cluster. The pairwise co-membership relationships from these LOSO assignments are then compared to those from the full clustering. The final score is the fraction of all pairs where the relationship agrees, ranging from 0 (unstable) to 1 (identical structure with any participant removed).

Cluster predictability measures whether the cluster assignments reflect systematic patterns in the feature space rather than noise. A logistic regression classifier is trained on the cluster labels of all participants except one; then, it is asked to predict the held-out participant’s cluster. The score is the fraction of participants correctly predicted. High predictability indicates the clusters occupy distinct, learnable regions of the feature space, and low predictability suggests the cluster boundaries are arbitrary or driven by noise. These per-movement clustering results were used to build the coassociation matrices for final subgrouping.

### 3.7. Cross-Movement Subgroup Discovery

Ensemble clustering is a technique used to combine multiple clustering methods for improved results and can be conducted in many ways. We follow the evidence accumulation approach in this paper [61,62]. We formatted the per-movement clustering results for each k as an averaged coassociation matrix whose values represent the frequency of movements for which each pair of participants were assigned to the same cluster.

Next, we applied both hierarchical clustering and spectral clustering, which are well suited to determine clusters from such matrices, to determine subgroups. Spectral clustering models data points as a graph and uses graph theory and Laplacian eigenvalues to detect complex, non-convex clusters [18]. Hierarchical clustering constructs a nested hierarchy of clusters by iteratively grouping data points based on similarity [17].

We evaluated the results for *k* = 2, 3, 4, given our sample size of 10 and plausible number of LBP subgroups. We then characterize subgroups based on the top differentiating features and explore pain-related differences.

## 4. Results and Discussion

We begin this section with the experimental results involving time-series foundation models. We highlight their weaknesses for LBP subgrouping as a motivating factor for our proposed pipeline based on biomechanical and causal feature extraction. We then show the results of our approach including movement-specific and multiple-movement differentiating features, which we use to characterize LBP subgroups. Finally, we compare participant characteristics across subgroups.

The subgroups identified in this paper are exploratory based on skin-strain-derived movement and coordination patterns, and they should not be interpreted as validated clinical LBP subgroups. The confirmation of clinically distinct subgroups will require replication in larger cohorts of individuals with LBP.

### 4.1. Weaknesses of Time-Series Foundation Model Analysis

As a baseline reference, we assessed whether general-purpose time-series foundation models provide representations that capture LBP subgroup structure. MOMENT and Chronos were used to generate embeddings from our strain signals [63,64]. MOMENT (large) produced a 1024-dimensional embedding for each of the six sensor channels. These per-channel embeddings were mean-pooled, providing a single 1024-dimensional representation per participant per movement. Chronos (base) per-channel embeddings were mean-pooled as well to produce a single 768-dimensional representation per participant per movement.

We applied k-means and agglomerative clustering with *k* = 2, 3, 4 and calculated LOSO consistency and logistic regression predictability for the various methods (Appendix Table A1). They indicate that the clusterings are mostly stable. However, these metrics are less meaningful in high-dimensional embedding space for our sample size. Therefore, we also calculated the cosine distances between and within clusters.

**Figure 6:**
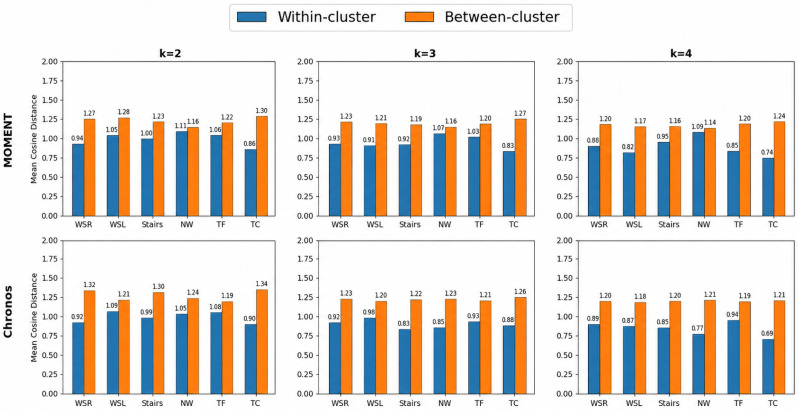
Within-cluster versus between-cluster mean cosine distance for MOMENT and Chronos embeddings across six movements (WSR = WeightedSuitcaseRight, WSL = WeightedSuitcaseLeft, NW = NormWalk, TF = TabletFloor, TC = TabletCabinet), using agglomerative clustering and three values of *k*. Cosine distance ranges from 0 (identical) to 2 (opposite) with 1.0 indicating orthogonality. Similar bar heights across all configurations indicate that subjects assigned to different clusters are no more distant in embedding space than subjects within the same cluster, suggesting weak cluster separation.

**Figure A1:**
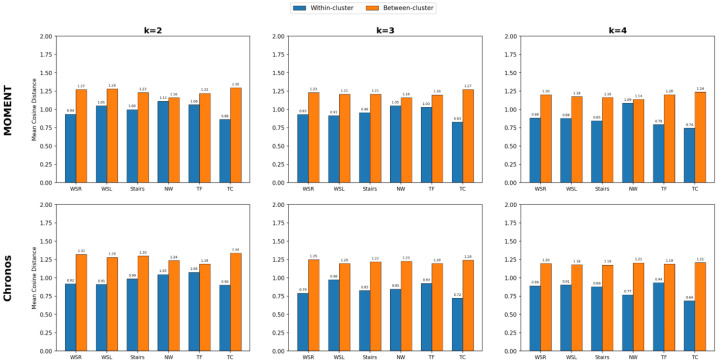
Within-cluster versus between-cluster mean cosine distance for MOMENT and Chronos embeddings across six movements (WSR = WeightedSuitcaseRight, WSL = WeightedSuitcaseLeft, NW = NormWalk, TF = TabletFloor, TC = TabletCabinet), using k-means clustering and three values of *k*. Cosine distance ranges from 0 (identical) to 2 (opposite) with 1.0 indicating orthogonality. Similar bar heights across all configurations indicate that subjects assigned to different clusters are no more distant in embedding space than subjects within the same cluster, suggesting weak cluster separation.

Cosine distance is a metric well suited for foundation model embeddings that measures if they point in similar directions in the high-dimensional embedding space. Notably, as shown in Figure 6 for agglomerative clustering results (and Appendix Figure A1 for k-means clustering results), for all movements and configurations, both within and between cluster cosine separation distances were fairly close to 1 (the value of 0 indicates perfect similarity and 2 indicates opposite embeddings). This means embeddings within the same cluster are similarly distant to embeddings in different clusters, suggesting weak separation between clusters. The embeddings likely capture general temporal patterns like signal shape, periodicity, and amplitude. Most importantly, they lack clinical interpretability with respect to LBP subgroups.

Next, the cross-movement ensemble clustering stage of our pipeline was used to discover LBP subgroups. There were significant differences between MOMENT and Chronos in cluster structure and membership. Ultimately, the final LBP subgroups were clinically uninterpretable as well, which we aim to improve with our approach based on feature extraction.

### 4.2. Movement-Specific Clustering Analysis Using Biomechanical and Causal Features

This section presents the clustering results using the feature extraction approach described in Section 3. The analysis is based on the six selected movements with the highest stationarity scores from Table 3. In summary, for each participant’s movement trial, we generated (1) five composite time series based on the six sensor time series, from which we extracted 20 biomechanical features (Table 2), and (2) a PCMCI+ causal graph with nodes as the six sensors and the contemporaneous or lagged links, from which we extracted 31 causal features (per-phase and cross-phase features from Table 5).

Separately for each movement, we used k-means and agglomerative clustering to examine the top features by effect size. For the *k* = 2 experiments, this was calculated using Cohen’s d, which represents the standardized difference between two groups. For *k* > 2, we used eta-squared values. Eta-squared is a measure of effect size that provides the percentage of variance in the dependent variable that is explained by the independent variable [65]. The complete results comparing k-means and agglomerative clustering top 10 features for each movement are in Appendix Table A2, Table A3, Table A4, Table A5, Table A6, Table A7.

The top 10 features were nearly identical for k-means and agglomerative clustering given the same movement and *k*, suggesting that the selected features are not merely artifacts of the clustering method. At *k* = 2, four of the six movements had perfect agreement between k-means and agglomerative clustering top features. For all values of *k*, the mean agreement between the two clustering methods on top features across all movements was approximately 80 percent. As in this section the feature importance is derived from clustering per-movement, the top features here may slightly differ from the top features that result from ensemble clustering across all movements; therefore, the features are described in detail in the next section.

Table 6 presents the results for LOSO cluster consistency (fraction from 0 to 1 of pairwise same-cluster relationships preserved when each participant is sequentially removed and reassigned) and cluster predictability (leave-one-out logistic regression accuracy from 0 to 1 on the cluster labels) for k-means and agglomerative clustering with *k* = 2, 3, 4 clusters. Most experiments showed high predictability and LOSO clustering consistency, indicating meaningful cluster structure [58]. Agglomerative clustering consistently outperformed k-means clustering in these metrics, suggesting it may be better suited for this dataset.

### 4.3. LBP Subgroup Characterization Across Movements

Our subgrouping experiments involved applying ensemble clustering to cluster the coassociation matrices formed by per-movement clustering [61,62]. We chose agglomerative clustering to use for this ensemble clustering because it outperformed k-means clustering with respect to consistency (as seen in Table 6). For each per-movement *k*, we start with the per-movement clustering results from agglomerative clustering and create the coassociation matrix representing the proportion of movements for which each pair of participants were assigned to the same cluster. Then, we used hierarchical and spectral clustering to determine LBP subgroups across movements (subgroup *k*). Across all methods and choices of *k* for final subgroups, minor differences in cluster assignments were observed, which is typical for limited sample sizes. Nevertheless, the results indicate an underlying substructure, which demonstrates that the subgroups are not artifacts of a specific clustering technique or k-selection.

**Figure 7:**
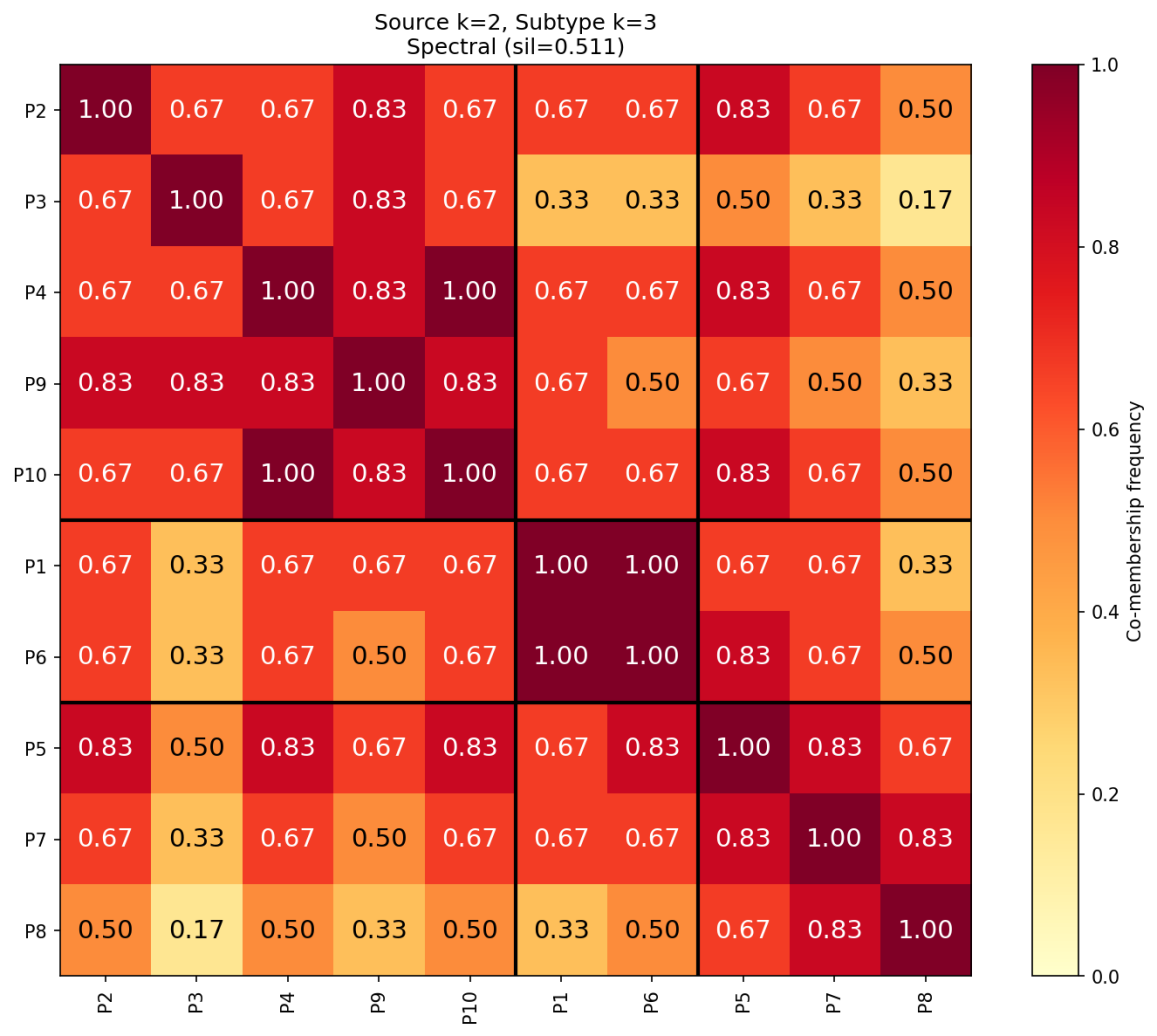
Heatmap of coassociation matrix representative of subgroups A (*n* = 2; P1, P6), B (*n* = 3; P5, P7, P8), C (*n* = 5; P2, P3, P4, P9, P10), where P1–P10 refer to participant numbers.

We identified three subgroups, which we named as subgroups A (*n* = 2), B (*n* = 3), and C (*n* = 5), respectively. This three-subgroup structure was produced with the per-movement *k* = 2 coassociation matrix using spectral clustering with LBP subgroup *k* = 3. Figure 7 displays the coassociation matrix as a heatmap of individual participants (P1–P10) sorted by subgroup, where darker cells indicate higher coassociation values.

Clustering quality is often evaluated by silhouette score. It reflects how similar each point is to its own cluster compared to other clusters, which is averaged across all points. The score ranges from −1 to 1; positive values near 1 indicate strong assignment to a cluster, values near 0 suggest points lie near cluster boundaries or overlap, and negative values imply possible misclassification [66]. While higher scores generally indicate better-defined clusters and traditionally a silhouette score over 0.5 is considered good, the interpretation depends on the dataset, feature space, and cluster geometry [66]. As a result, silhouette scores are most often used comparatively, for example, to select the optimal number of clusters. Clusterings that best capture the underlying subgroup structure tend to achieve higher silhouette scores.

Table 7 presents the silhouette scores of all experiments, and those supporting our subgroups are indicated. Rather than selecting subgroups from a single configuration, we evaluated all combinations of per-movement *k* (2, 3, 4), subgroup *k* (2, 3, 4), and two clustering methods (hierarchical and spectral). The reported three-subgroup structure was selected because it achieved among the highest silhouette scores and was corroborated across multiple configurations. Thus, the *k* = 3 solution was preferred over *k* = 2, which merged subgroups with distinct movement characteristics into a single cluster and had lower silhouette score; and it also was preferred over *k* = 4, which produced additional splits that were less reproducible across configurations.

The three subgroups were obtained with per-movement *k* = 2, LBP subgroup *k* = 3 spectral clustering (silhouette = 0.511). Additional support came from the per-movement *k* = 2, LBP subgroup *k* = 4 spectral clustering (silhouette = 0.517), which recovered subgroups A and B exactly while splitting the larger subgroup C into two smaller subgroups containing two and three participants, respectively. The hierarchical method at per-movement *k* = 2 consistently recovered the two participants in subgroup A as a distinct pair across subgroups *k* = 3 and *k* = 4 (silhouette = 0.516 and 0.472), though it placed one participant from subgroup B into the larger subgroup C. Subgroup B was further supported at per-movement *k* = 3 and *k* = 4, where the same three-participant subgroup emerged as a cluster in multiple spectral solutions. There was one participant who was considered a borderline subject, whose placement shifted between B and C depending on the configuration.

Subgroup differences were characterized using $\eta^{2}$, which quantifies the proportion of variance in each feature explained by subgroup membership. The top discriminating features of the subgroups for each movement (see Appendix Table A8) were broadly consistent with the biomechanical demands of the task. For lateral bending tasks (WeightedSuitcaseRight and WeightedSuitcaseLeft), the dominance of left–right asymmetry and lateral range features aligns with the asymmetric primary phase demands of the movement. For stair climbing, the prominence of causal sensor net influence features is consistent with the coordination demands of ascending and descending stairs, which may be affected by LBP and require compensatory strategies. For normal walking, the relatively weaker feature discrimination is not surprising, as level walking is a highly practiced, low-demand task that may not be challenging for participants with LBP in this paper. For TabletFloor, the strong discrimination by return-phase causal features indicates that the return from a forward bend position is where individuals with LBP commonly exhibit altered patterns of movement. The dominance of range and velocity features in TabletFloor and TabletCabinet reflect the large trunk excursions required, which amplify individual differences in movement strategy that may be less visible during lower-demand activities.

Table 8 shows features that appeared in the top 10 discriminating features (by $\eta^{2}$) for at least two movements when comparing the subgroups. The overall range and maximum rate of change were the most common discriminators, appearing in the top 10 for all six movements with moderate to strong mean effect sizes. The return phase sensor 6 (lower right) net influence and the return phase contemporaneous diagonal ratio were the most consistent causal features, particularly for bending tasks, reflecting subgroup differences during the return phase.

Subgroup A (P1, P6) demonstrated asymmetric behavior with distinctive coordination. Subgroup A consistently showed the largest left-side sensor strain range across movements (highest in four of six tasks) and the highest positive left–right asymmetry in many movements, indicating greater trunk strain on the left side during diverse tasks. During flexion, subgroup A exhibited extreme values in causal per-sensor net influence at the lower-right sensor 6 during return (TabletFloor, 0.94), a pattern not seen in subgroups B or C, suggesting a distinctive inter-sensor coordination strategy during the return to upright position. Causal per-sensor net influence features for the upper-left sensor 1 during both primary and return phases further distinguished subgroup A during stair climbing and lateral bending movements, suggesting a distinct coordination strategy.

Subgroup B (P5, P7, P8) can be characterized by the participants’ overall large-amplitude strain signals. Subgroup B consistently produced the largest overall sensor strain range during flexion tasks (TabletFloor and TabletCabinet) and the greatest right-side strain during both flexion, lateral bending, and stair climbing. During normal walking, B showed the most positive return-phase left–right causal asymmetry (0.56) and the largest overall strain range. This suggests subgroup B might have less restricted movement resulting in the greatest amplitudes of strain overall.

Subgroup C (P2, P3, P4, P9, P10), the largest subgroup, can be described as a low strain amplitude, overall moderate movement group, which may reflect limited mobility. Subgroup C consistently showed the lowest amplitude strains during trunk displacement and the most symmetrical primary phase pattern across all tasks. The overall range in subgroup C was lower than A and B, and left–right asymmetry in subgroup C was near zero or mildly negative, contrasting with A’s strong positive values. During normal walking, subgroup C shows a significant phase-dependent shift in left–right contemporaneous causal linking (*p* = 0.04), indicating a normal lateral reversal in walking and more symmetric movement overall compared to the other subgroups. In terms of causal features, the return phase proportion of contemporaneous diagonal links tended to be highest in subgroup C during lateral bending and flexion tasks, suggesting a more globally coupled return-to-upright strategy rather than largely horizontal or vertical. This indicates subgroup C may return to upright as a more unified segment, which is consistent with a guarded movement pattern.

**Figure 8:**
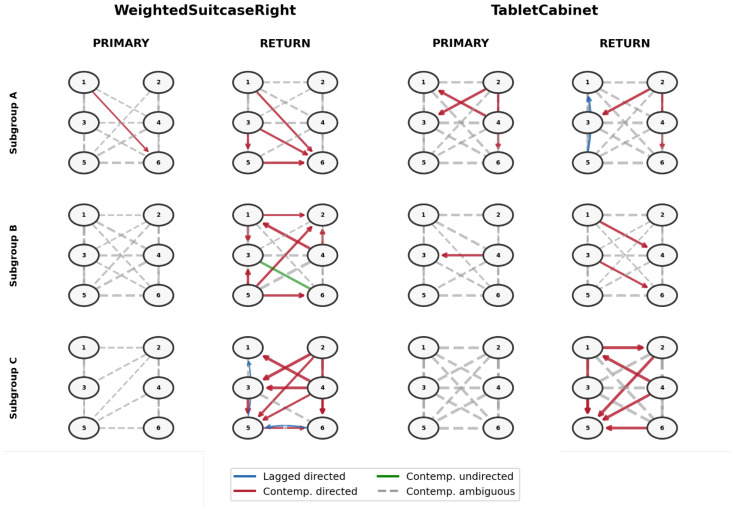
Causal relationship graphs of primary and return phases of WeightedSuitcaseRight (lateral bending) and TabletCabinet (flexion) movements for representative subjects from each subgroup. The numbers 1–6 refer to Sensors 1–6 from Figure 1.

Figure 8 shows causal relationship graphs for both phases of two movement tasks, WeightedSuitcaseRight and TabletCabinet, for a single participant from each subgroup, providing a visual representation of some of the PCMCI+ derived features. Contemporaneous directed, lagged directed, contemporaneous undirected, and contemporaneous ambiguous edge types are visually differentiated. The dominance of contemporaneous edges makes intuitive sense given the approximately 17 ms sampling rate during continuous movement, as causal influences propagate faster than the inter-sample gap and appear simultaneous. Subgroup A had the most directed edges going toward the lower-right sensor 6 in both of the TabletCabinet and WeightedSuitcaseRight return phase graphs, which was reflective of the group’s’ unique asymmetric coordination strategy (most negative return phase sensor 6 net influence). Subgroup C’s TabletCabinet and WeightedSuitcaseRight graphs show the highly coupled diagonal sensors in the return phase discussed earlier. Subgroup B is largely characterized by overall amplitude and less by specific causal features in these movements, which we can see by its comparatively sparser graph (TabletCabinet) or overall similarity (WeightedSuitcaseRight).

Overall, subgroups are distinguished both by high-level biomechanical features (such as range and rate of change) and spatial coordination strategies (indicated by features such as asymmetry, dominant sensor regions, and contemporaneous linked sensor patterns), as described in Table 9. The majority of participants with LBP (subgroup C) demonstrated moderate restrained trunk movement across tasks, whereas the other two smaller subgroups diverged in distinct ways, one through pronounced lateral asymmetry (subgroup A) and the other through higher overall strain amplitudes (subgroup B).

### 4.4. Clinical LBP Characteristics by Subgroups

We next analyze the self-reported clinical characteristics of each subgroup. These variables were not used during clustering, as our goal was to perform unsupervised clustering based on objective sensor streams. However, they offer additional insights about the subgroups. Subgroup A had the shortest average LBP duration (both members with duration between 1–5 years). All three subgroups included individuals who experienced pain while sitting, standing, and walking. These exploratory observations are not meant for causal interpretation and require assessment in larger samples. Additionally, because these characteristics were noted only during the trials, variables that change over time may not be representative of an individual’s history of LBP.

We further provide an exploratory analysis of participants’ self-reported pain increase during each movement, as shown in Table 10. Negative numbers indicate a decrease in pain compared to the baseline position. Subgroup A had the highest mean pain increase during the WeightedSuitcase right/left lateral bending movements, which is expected considering the group’s lateral asymmetry. Subgroup A also had the highest reported mean pain for TabletCabinet. Subgroup B reported minimal pain increase overall, which is not surprising as subgroup B had higher strain values, which indicated more movement overall or simply that pain was not provoked by these tasks. This finding would need to be confirmed in a larger sample. Within subgroup C, the highest mean pain was reported during the TabletFloor and TableCabinet movements. Across all participants in all subgroups, the TabletFloor and TableCabinet tasks were most frequently associated with an increase in pain. Also, only one participant reported any increase in pain during NormWalk, suggesting level walking is well tolerated in this LBP cohort. These patterns are preliminary given the small sample size.

## 5. Limitations and Opportunities for Future Work

We present these findings as an exploratory analysis of the capability of MT technology and our analytic framework for LBP subgrouping based on movement and coordination strategies. We recognize that the main limitation of this paper is the small sample size of 10 participants. This is due to the limited availability of the newly developed MT sensor technology when we conducted this research. Nevertheless, testing an extensive set of movements, including functional tasks and sampling at 60 Hz, a suite of six sensors provide significant data points for movement analysis.

We addressed data quality in every step of our pipeline, beginning with data acquisition. A physical therapist selected the movements to be tested to ensure they are clinically relevant for LBP assessment. Sensors were placed consistently by the same investigator trained by the physical therapist. Our protocol was to place the middle MTs in a standard anatomic location and then place the superior and inferior MTs adjacent to and aligned with the middle MTs. The consistency of inter-sensor distances was ensured by having the k-tapes directly adjacent to one another without overlapping and the MWCNT sensing regions in the same relative location on the tapes. This means that depending on each individual’s anatomy, the sensing regions of the upper and lower tapes may not be in exactly the same anatomic location. However, these differences among people are very minimal.

In addition, MT resistance can drift over time. To address this, MT baselines were recorded prior to each movement trial, and signal values were normalized to the baselines. Furthermore, data collection was conducted in a controlled setting over a limited period of time (two hours per participant including lab setup). Future studies involving monitoring participants in free-living environments will need to mitigate sensor drift.

To avoid movement-specific bias, we selected a range of tasks spanning multiple planes of motion. Feature engineering, particularly for causal features, involved deliberate methodological choices. The sensor sampling rate (60 Hz) provided dense time-series data, and the selection of movements that satisfied stationarity assumptions while retaining sufficient data length created favorable conditions for applying PCMCI+. This algorithm was selected for its suitability to such data, including an improved handling of autocorrelation and enhanced detection of adjacency relationships as well as lagged and contemporaneous links.

PCMCI+ performs conditional independence testing across all timesteps assigned to a given phase simultaneously. If the underlying causal structure changes partway through the time series, the resulting causal graph could be affected. Although our stationarity assessment supported approximate within-phase stability for the selected movements, subtle non-stationarity, such as gradual shifts in coordination due to fatigue, cannot be fully ruled out. To illustrate, consider a scenario in which sensor 1 causally drives sensor 5 during the return phase of early repetitions, but fatigue leads the subject to shift strategy in later repetitions such that sensor 3 causally drives sensor 5 instead. Because PCMCI+ pools all repetitions, the sensor 1→5 partial correlation is attenuated and therefore lower than it would appear if only the early repetitions were analyzed, but it is possibly still above the significance threshold. The sensor 3→5 link is similarly diminished. As a result, PCMCI+ may output both links at reduced strength, miss both if neither pass the significance test, or retain only whichever link was stronger overall. However, our movement testing was performed in a controlled environment in the lab, and there were only three repetitions for each movement to limit variations due to fatigue.

The small number of participants and the high ratio of features to participants may lead to overfitting, but there are several considerations that mitigate this concern in our approach. The context of overfitting differs in unsupervised clustering compared to supervised prediction; here, there are no labels being fit, and the risk is that clusters may capture noise structure in the feature space rather than meaningful subgroups. To address this concern, in the per-movement clustering stage, we evaluated cluster robustness using metrics such as LOSO stability and logistic regression-based predictability to ensure that cluster assignments were not driven by individual participants. Stability was assessed based on the assumption that meaningful clusters persist across small perturbations of the data [60]. Agglomerative clustering achieved a mean LOSO consistency of 0.75–0.84 across k values and mean predictability of 0.69–0.84, indicating that cluster assignments were not driven by individual participants and that the clusters occupy systematically distinct regions of the feature space. We also observed agreement across clustering methods in identifying key differentiating features. Post hoc analysis of the top discriminating features confirmed that the features driving subgroup separation were clinically interpretable and consistent with the known biomechanical demands of each movement type, suggesting the subgroup structure is not an artifact of noise fitting. Furthermore, the ensemble clustering framework provides an additional layer of robustness as subgroups are derived not from a single clustering of 51 features but from coassociation matrices that aggregate clustering results across six independent movements. At the cross-movement level, consistent subgroup structures were observed across clustering algorithms and choices of k, further supporting the validity of the identified subgroups and suggesting they are not artifacts of specific methodological choices.

The limited sample size is typical at this phase of sensor development, as analysis is intended to guide sensor refinements prior to larger-scale studies. Still, this restricts the generalizability of the three LBP subgroups to broader LBP populations, which may include more or different subgroups. Nevertheless, the experimental results provide unique insights in LBP movement patterns supported by MT. Notably, due to the novelty of the MT technology, external datasets are not yet available for validation. Also, clinical ground truth labels for LBP subgroups are not available for this cohort; in fact, subgrouping is challenging due to the difficulty of objectively assessing fine-grained movement and strain on the spine, which is exactly what MT shows promise in addressing. Accordingly, we present these results as exploratory, providing early insights into movement stationarity, informative features, limitations of time-series foundation models for clinical interpretation, and a preliminary LBP subgrouping framework. Furthermore, the methodology based on biomechanical and feature extraction is an important contribution in itself and it could be extended to other sensor modalities.

In future studies with a larger sample size and more movements, several improvements are possible. Finer-grained subgroups may emerge that capture different movement-specific and cross-movement coordination strategies. Additionally, a larger cohort may provide better insight into an optimal number of subgroups. Correlation analysis between specific features and pain increase could reveal how movement and coordination patterns are associated with pain responses. Furthermore, more participants are needed with a variety of body types, as it is important to evaluate how MT signals vary depending on body composition. For the current cohort, variation was relatively modest (i.e., height: 172.1 ± 7.3 cm, weight: 80.7 ± 23.2 kg, waist–hip ratio: 0.83 ± 0.07), which limits the extent to which body habitus effects could confound our subgroup findings. Therefore, a systematic characterization of how body composition influences MT amplitude and timing features is an important direction for future work with larger and more diverse cohorts.

The clinical motivation for identifying LBP subgroups using MT data is that individuals with LBP are heterogeneous in how they move, and identifying distinct coordination profiles could inform tailored treatment approaches. For example, a patient with LBP who belongs to the subgroup exhibiting lateral asymmetry may benefit from different interventions than a patient in the subgroup exhibiting globally restrained movement. The relative differences between subgroups can be informative for guiding treatment decisions. This perspective aligns with LBP literature advising subgroup-specific treatment rather than one-size-fits-all approaches [3,33]. Ultimately, this information could guide the development of movement-based interventions tailored to each subgroup’s movement profile, for instance addressing asymmetric movement in some subgroups, reducing excessive strain exposure in others, and encouraging greater movement within pain and strain tolerance in those who may be compensating through restriction.

Future studies comparing the MT strain profiles of people with LBP to a healthy control group could provide additional clinical context and could help determine which subgroup-specific patterns reflect pain-related adaptations. Additional analysis could identify impairments from comparing the movements between people with and without LBP, determining how movements are associated with LBP progression, estimating the cumulative strain on the lower back, and assessing the risk of LBP or recurrence.

## 6. Conclusions

This paper is an exploratory investigation of whether MT data can be used to discover movement-based LBP subgroups that could be clinically meaningful. We engineered causal features from PCMCI+ and biomechanical signal features and found that these complementary feature sets provide unique insights into how movement patterns in LBP subgroups differ and demonstrate the importance of a diverse movement set when examining people with LBP.

The comparison with foundational time-series models shows that general-purpose time series representations can capture some structure though with fairly weak separation between clusters. However, more importantly for movement analysis in people with LBP, it is unclear what the clusters represent and what kinds of signal qualities the model is recognizing, supporting the value of domain-specific feature engineering for clinical subgrouping applications.

The use of MT technology for a low back use case with well-defined sensor placement enabled us to identify three subgroups that differ in specific coordination strategies and overall movement. Specifically, we observed a predominant LBP subgroup exhibiting moderate trunk strain across tasks and two smaller distinctive subgroups characterized by lateral asymmetry and greater overall strain. Although these initial findings require further investigation given the sample size, they show that MT and feature engineering provide a promising technology for LBP subgrouping analysis.

## Figures and Tables

**Table 1:** Movements for human subjects testing in the lab.

Category	Position	Movement	Direction
Clinical movement tasks	Standing	Forward flexion	Flexion
		Extension	Extension
	Sitting	Rotation left/right	Rotation
Driving tasks	Sitting	Driving (5 min)	Flexion
		Reverse driving left/right	Rotation
Pickup functional tasks	Standing	Picking up tablet from floor	Flexion
		Placing tablet in cabinet	Flexion
		Picking up suitcase left/right	Lateral bending
		Picking up weighted suitcase left/right	Lateral bending
	Sitting	Picking up bottle (left, right)	Lateral bending
Additional functional tasks	Standing	Hanging a towel	Extension
		Putting in lightbulb	Extension
		Stair climbing (10 stairs up and down)	N/A
		Normal walking (35 feet)	N/A
		Downhill walking (≈5% incline)	N/A

**Table 2:** Biomechanical features. A total of 20 features are extracted per subject per movement.

Feature	Definition	Interpretation
Computed for each composite (top, bottom, left, right, overall) → 15 features		
Range	$x_{\max}-x_{\min}$	Extent of lumbar spine strain during the movement
Max rate of change	$\max_{i}\left|x_{i+1}-x_{i}\right|\cdot f_{s}$	Captures the most rapid signal change during the movement (strain rate during primary or return phase)
Mean near-peak duration	Mean duration of contiguous segments where $|x_{i}|\geq 0.95\cdot \max|x|$	Mean time spent at near-maximal strain
Computed from overall composite (magnitude) → 3 features		
Jerk	$\frac{\sqrt{\frac{1}{N}\sum_{i}{\left(\frac{d^{3}x}{dt^{3}}\right)}_{i}^{\;2}}}{R\cdot T}$, where *R* = range, *T* = duration. Signal low-pass filtered at 6 Hz prior to differentiation.	Normalized jerk as a measure of movement smoothness; higher values indicate jerkier, less controlled movement
Peak amplitude CV	$\frac{\sigma (A_{\mathrm{peaks}})}{{\bar{A}}_{\mathrm{peaks}}}$, where $A_{\mathrm{peaks}}$ are the peak amplitudes of the magnitude signal	Coefficient of variation of peak strain amplitudes across repetitions; higher values indicate inconsistency across repetitions
Inter-peak interval CV	$\frac{\sigma (\Delta t_{\mathrm{peaks}})}{{\bar{\Delta t}}_{\mathrm{peaks}}}$, where $\Delta t_{\mathrm{peaks}}$ are the inter-peak intervals in seconds	Coefficient of variation of timing between consecutive peaks; higher values indicate irregular timing between repetitions
Computed from left/right and top/bottom composite pairs → 2 features		
Left–right asymmetry	$\frac{{\bar{x}}_{L}-{\bar{x}}_{R}}{|{\bar{x}}_{L}\left|+\right|{\bar{x}}_{R}|}$	Normalized bilateral difference in mean strain; higher values reflect more left-column sensor strain
Top–bottom asymmetry	$\frac{{\bar{x}}_{T}-{\bar{x}}_{B}}{|{\bar{x}}_{T}\left|+\right|{\bar{x}}_{B}|}$	Normalized vertical difference in mean strain; higher values reflect more top-row sensor strain

**Table 3:** Within-phase stationarity assessment across candidate movements. Movements above the line were selected for subsequent analysis. Phases were rated as: good/adequate if correlation shift < 0.25 and similarity > 0.6; marginal if correlation shift ≥ 0.25 or similarity ≤ 0.6. Mean *n*/phase is the average number of timesteps per phase in a subject’s movement sample averaged across both phases (primary and return) and across all subjects for that movement.

Movement	Stationarity	Good or	Marginal	Mean *n*/
	Score	Adequate (%)	(%)	Phase
Picking up weighted suitcase right	0.94	94.4	5.6	462
Picking up weighted suitcase left	0.78	77.8	22.2	432
Stair climbing (up and down)	0.65	65.0	35.0	980
Normal walking	0.61	61.1	38.9	514
Picking up tablet from floor	0.60	60.0	40.0	581
Placing tablet in cabinet	0.60	60.0	40.0	597
Reverse driving left	0.52	55.6	44.4	244
Downhill walking	0.48	60.0	40.0	156
Hanging a towel	0.43	50.0	50.0	264
Rotation right	0.40	50.0	50.0	180
Rotation left	0.38	50.0	50.0	166
Forward flexion	0.33	33.3	66.7	230
Putting in lightbulb	0.28	33.3	66.7	429
Extension	0.16	22.2	77.8	195
Reverse driving right	0.15	16.7	83.3	204

**Table 4:** Descriptions of movements selected for analysis. All movements were performed standing.

Movement	Description
Lateral Bending Functional Tasks	
Picking up weighted suitcase left/right (WeightedSuitcase)	Bending to the left (right) to pick up a weighted suitcase (10 lb) with your left (right) hand, return to upright, then return the suitcase to the floor and return to upright.
Flexion Functional Tasks	
Picking up tablet from floor (TabletFloor)	Bending forward to place a tablet on the floor and then return to upright; instructed to use the back and hips but may bend slightly in the knees if needed.
Placing tablet in cabinet (TabletCabinet)	Bending forward to place a tablet in a lower cabinet, return to upright, then bend forward to retrieve the tablet from the cabinet and return to upright.
Gait Tasks	
Stair climbing up and down (Stairs)	Walking up 10 stairs to the top of the staircase, pause and turn around, then walk down the same 10 steps; may lightly touch the handrail for balance if needed.
Normal walking (NormWalk)	Walking on a flat straight sidewalk at a normal pace. Walk from one marked line to another in a straight direction and return to rest.

**Table 5:** Features derived from PCMCI+ causal discovery results.

Feature	Definition	Interpretation
Per-phase features (computed for primary and return separately)		
$n_{\mathrm{contemp}}$	Count of detected contemporaneous links	Higher values may may reflect more strain propagation between regions
Contemp. link ratios	$\frac{n_{c}}{n_{\mathrm{contemp}}}$ for c∈{horiz,vert,diag}	Captures whether detected contemporaneous links are predominantly bilateral, vertical along the spine, or cross-body; reflective of main strain propagation direction
Per-sensor net influence ($S_{k}$_net)	$\mathrm{out}\left(S_{k}\right)-\mathrm{in}\left(S_{k}\right)$ where out = number of directed links originating from $S_{k}$, in = number directed toward $S_{k}$	Net causal influence per sensor; captures if underlying segment’s strain mainly leads (positive) or follows (negative) that of other segments during the phase. Computed for each sensor $S_{1}$–$S_{6}$.
left–right causal asymmetry	$\frac{{\mathrm{out}}_{L}-{\mathrm{out}}_{R}}{\max({\mathrm{out}}_{L}+{\mathrm{out}}_{R},\;1)}$	Lateral distribution of outgoing causal influence; positive = left-dominant, negative = right-dominant
Top–bottom causal asymmetry	$\frac{{\mathrm{out}}_{T}-{\mathrm{out}}_{B}}{\max({\mathrm{out}}_{T}+{\mathrm{out}}_{B},\;1)}$	Vertical distribution of outgoing causal influence; positive = upper-dominant, negative = lower-dominant
Diagonal vs. vertical ratio	$\frac{n_{\mathrm{diag}}^{\to }-n_{\mathrm{vert}}^{\to }}{\max(n_{\mathrm{diag}}^{\to }+n_{\mathrm{vert}}^{\to },\;1)}$	Balance of cross-body and same-column directed causal pathways; positive = diagonal-dominant coordination, negative = vertical-dominant
Cross-phase features (primary vs. return)		
Phase contemp. fraction diff	$|f_{\mathrm{primary}}^{c}-f_{\mathrm{return}}^{c}|$ for c∈{horiz,vert,diag}	Captures phase-dependent changes in coordination topology
Phase left–right asymmetry diff	$|{\mathrm{LR}}_{\mathrm{primary}}-{\mathrm{LR}}_{\mathrm{return}}|$	Magnitude of lateral coordination reversal between phases; high values indicate primary and return are driven by opposite sides
Phase top–bottom asymmetry diff	$|{\mathrm{TB}}_{\mathrm{primary}}-{\mathrm{TB}}_{\mathrm{return}}|$	Magnitude of vertical coordination reversal between phases; high values indicate upper–lower dominance shifts between primary and return

**Table 6:** Per-movement clustering performance metrics. Consistency = LOSO Clustering Consistency, Predictability = LOSO LogReg Predictability.

		KMeans		Agglomerative	
Movement	k	Consistency	Predictability	Consistency	Predictability
WeightedSuitcaseRight	2	0.44	0.89	1.00	0.89
	3	0.67	0.89	0.89	0.89
	4	0.64	0.78	0.81	0.78
WeightedSuitcaseLeft	2	0.50	0.78	0.78	0.78
	3	0.72	0.67	1.00	0.78
	4	0.75	0.78	0.86	0.78
Stairs	2	0.53	0.90	0.80	0.90
	3	0.47	0.70	0.80	0.70
	4	0.58	0.70	0.73	0.60
NormWalk	2	0.50	0.78	0.50	0.78
	3	0.53	0.78	0.81	0.78
	4	0.53	0.67	0.56	0.67
TabletFloor	2	0.47	0.80	0.64	0.80
	3	0.67	0.60	0.73	0.60
	4	0.62	0.60	0.87	0.60
TabletCabinet	2	0.80	0.90	0.80	0.90
	3	0.47	0.80	0.80	0.70
	4	0.58	0.50	0.98	0.70
Mean across movements	2	0.54	0.84	0.75	0.84
	3	0.59	0.74	0.84	0.74
	4	0.62	0.67	0.80	0.69

**Table 7:** Silhouette scores for subgrouping from agglomerative clustering coassociation matrices. Per-movement *k* refers to the number of clusters used in the per-movement clustering step that produces the coassociation matrices, and subgroup *k* refers to the number of LBP subgroups extracted by clustering those matrices. **Bold** = exact match to reported subgroups A, B, C. ^†^ = partial support (key pairs preserved within larger clusters).

Per-Movement *k*	Subgroup *k*	Hierarchical	Spectral
2	2	0.390 ^†^	0.346 ^†^
	3	0.516 ^†^	**0.511**
	4	0.472 ^†^	0.517 ^†^
3	2	0.330 ^†^	0.330 ^†^
	3	0.330	0.370 ^†^
	4	0.383 ^†^	0.206 ^†^
4	2	0.241	0.264
	3	0.176	0.194 ^†^
	4	0.113	0.238

**Table 8:** Cross-movement feature consistency. Features appearing in top 10 ($\eta^{2}$) for ≥2 movements, ranked by number of movements (#Mvs). B/C = biomechanical feature (Table 2) or causal feature (Table 5).

Feature	B/C	#Mvs	$\bar{\eta^{2}}$	Movements
Overall range	B	6	0.73	WSR, WSL, Stairs, NW, TF, TC
Overall max rate of change	B	6	0.59	WSR, WSL, Stairs, NW, TF, TC
Right range	B	5	0.56	WSL, Stairs, NW, TF, TC
Left range	B	4	0.86	WSR, WSL, TF, TC
Return per-sensor net influence Sensor 6	C	4	0.71	WSR, NW, TF, TC
Left–right asymmetry	B	4	0.51	WSR, WSL, Stairs, TC
Left max rate of change	B	3	0.68	WSR, WSL, TC
Right max rate of change	B	3	0.47	WSL, Stairs, TC
Return contemp. coupling ratios diag.	C	2	0.67	WSR, TF
Primary per-sensor net influence Sensor 1	C	2	0.54	WSR, Stairs
Top mean near-peak duration	B	2	0.45	WSL, NW

WSR = WeightedSuitcaseRight, WSL = WeightedSuitcaseLeft, NW = NormWalk, TF = TabletFloor, TC = TabletCabinet.

**Table 9:** Summary of LBP subgroups by defining features.

Subgroup	Characteristic	Supporting Features
Subgroup A (*n* = 2)	Lateral asymmetry	Largest left-side strain range (highest in 4/6 tasks) Highest positive left–right asymmetry across tasks Distinctive causal net influence at sensor 6 during return phase of flexion and lateral bending Highest primary-phase top–bottom asymmetry during lateral bending Distinctive causal net influence at sensor 1 during stair climbing and lateral bending
Subgroup B (*n* = 3)	Large overall displacement	Largest overall strain range during flexion tasks Greatest right-side strain during flexion, lateral bending, and stair climbing Most positive return-phase left–right causal asymmetry during walking Largest overall strain range during walking
Subgroup C (*n* = 5)	Moderate, restrainedmovement	Smallest lumbar strain amplitude and most symmetrical primary phase pattern across tasks Near-zero left–right asymmetry in terms of mean strain Only subgroup with significant phase-dependent shift in left–right causal asymmetry during walking, suggesting symmetric lateral coordination Highest contemp. diagonal and vertical coupling fractions during return phase of lateral bending and flexion, suggesting a denser inter-sensor coupling consistent with a more rigid strategy

**Table 10:** Pain increase (on 0–10 numeric pain rating scale) by subgroup per movement. Mean reported per subgroup.

Movement	Subgroup A (*n* = 2)	Subgroup B (*n* = 3)	Subgroup C (*n* = 5)
WeightedSuitcaseRight	2.00	0.33	0.40
WeightedSuitcaseLeft	1.00	0.00	0.20
Stairs	0.50	0.33	0.20
NormWalk	0.00	0.00	0.40
TabletFloor	0.50	−0.33	1.40
TabletCabinet	1.50	0.00	1.00

## Data Availability

The de-identified dataset used in this paper is available upon appropriate request to the corresponding author. The data are not publicly available, owing to ethical concerns.

## Appendix A

**Table A1:** Foundation model per-movement clustering performance metrics (mean ± SD across 6 movements). Sil = silhouette score, LOSO = leave-one-subject-out consistency, Bal = size balance (min/max cluster size), Pred = LOSO logistic regression predictability, CosSep = cosine separation ratio.

	Method	Sil	Consistency	Bal	Pred	CosSep
k=2						
MOMENT	KMeans	0.217±0.104	0.81±0.23	0.38±0.30	0.93±0.05	10.25±0.16
	Agglomerative	0.217±0.104	0.97±0.08	0.38±0.30	0.93±0.05	10.25±0.16
Chronos	KMeans	0.230±0.055	0.64±0.22	0.40±0.24	0.89±0.12	10.32±0.15
	Agglomerative	0.248±0.041	0.93±0.10	0.28±0.16	0.93±0.05	10.27±0.17
k=3						
MOMENT	KMeans	0.136±0.072	0.54±0.12	0.18±0.03	0.77±0.13	10.29±0.15
	Agglomerative	0.138±0.069	0.86±0.13	0.21±0.10	0.79±0.10	10.29±0.16
Chronos	KMeans	0.137±0.024	0.56±0.09	0.42±0.21	0.83±0.11	10.46±0.18
	Agglomerative	0.151±0.034	0.87±0.07	0.27±0.14	0.84±0.09	10.37±0.10
k=4						
MOMENT	KMeans	0.105±0.063	0.66±0.09	0.30±0.18	0.66±0.11	10.38±0.20
	Agglomerative	0.100±0.063	0.72±0.21	0.23±0.05	0.66±0.11	10.35±0.21
Chronos	KMeans	0.129±0.040	0.66±0.08	0.26±0.06	0.65±0.18	10.43±0.19
	Agglomerative	0.127±0.043	0.84±0.11	0.27±0.07	0.65±0.20	10.45±0.18

**Table A2:** Feature importance for WeightedSuitcaseRight. Effect size: Cohen’s *d* for k=2, $\eta^{2}$ for k>2.

Rank	KMeans		Agglomerative	
	Feature	Score	Feature	Score
k=2: Effect size (Cohen’s *d*)				
1	top_range	4.34	top_range	4.34
2	all_max_rate_of_change	3.06	all_max_rate_of_change	3.06
3	tb_asymmetry	2.79	tb_asymmetry	2.79
4	left_max_rate_of_change	2.78	left_max_rate_of_change	2.78
5	top_max_rate_of_change	2.25	top_max_rate_of_change	2.25
6	left_range	2.20	left_range	2.20
7	all_range	2.19	all_range	2.19
8	primary_lr_asymmetry	2.15	primary_lr_asymmetry	2.15
9	bottom_range	2.11	bottom_range	2.11
10	right_mean_near_peak_dur	2.00	right_mean_near_peak_dur	2.00
k=3: Effect size ($\eta^{2}$)				
1	return_contemp_diag_frac	0.97	return_contemp_diag_frac	0.97
2	phase_contemp_vert_frac_diff	0.93	phase_contemp_vert_frac_diff	0.93
3	primary_contemp_vert_frac	0.92	primary_contemp_vert_frac	0.92
4	return_contemp_vert_frac	0.89	return_contemp_vert_frac	0.89
5	primary_contemp_diag_frac	0.87	primary_contemp_diag_frac	0.87
6	primary_contemp_lr_frac	0.85	primary_contemp_lr_frac	0.85
7	top_range	0.83	top_range	0.83
8	all_max_rate_of_change	0.78	all_max_rate_of_change	0.78
9	return_n_contemp	0.77	return_n_contemp	0.77
10	peak_amplitude_cv	0.74	peak_amplitude_cv	0.74
k=4: Effect size ($\eta^{2}$)				
1	return_contemp_diag_frac	0.98	return_contemp_diag_frac	0.98
2	top_max_rate_of_change	0.97	top_max_rate_of_change	0.97
3	phase_contemp_vert_frac_diff	0.93	phase_contemp_vert_frac_diff	0.93
4	primary_contemp_vert_frac	0.92	primary_contemp_vert_frac	0.92
5	all_range	0.91	all_range	0.91
6	top_range	0.90	top_range	0.90
7	return_contemp_vert_frac	0.89	return_contemp_vert_frac	0.89
8	primary_contemp_diag_frac	0.87	primary_contemp_diag_frac	0.87
9	primary_contemp_lr_frac	0.86	primary_contemp_lr_frac	0.86
10	return_n_contemp	0.85	return_n_contemp	0.85

**Table A3:** Feature importance for WeightedSuitcaseLeft. Effect size: Cohen’s *d* for k=2, $\eta^{2}$ for k>2.

Rank	KMeans		Agglomerative	
	Feature	Score	Feature	Score
k=2: Effect size (Cohen’s *d*)				
1	phase_tb_asymmetry_diff	3.51	phase_tb_asymmetry_diff	2.83
2	return_S3_net	2.03	primary_S1_net	2.65
3	phase_lr_asymmetry_diff	1.85	primary_S3_net	2.65
4	top_max_rate_of_change	1.76	primary_lr_asymmetry	2.65
5	top_range	1.65	primary_tb_asymmetry	2.65
6	return_tb_asymmetry	1.24	primary_diag_vs_vert	2.65
7	return_contemp_lr_frac	1.02	interval_cv	2.01
8	bottom_mean_near_peak_dur	1.01	all_smoothness	1.88
9	bottom_max_rate_of_change	0.98	return_S4_net	1.68
10	peak_amplitude_cv	0.98	return_tb_asymmetry	1.66
k=3: Effect size ($\eta^{2}$)				
1	primary_S1_net	1.00	primary_S1_net	1.00
2	primary_S3_net	1.00	primary_S3_net	1.00
3	primary_lr_asymmetry	1.00	primary_lr_asymmetry	1.00
4	primary_tb_asymmetry	1.00	primary_tb_asymmetry	1.00
5	primary_diag_vs_vert	1.00	primary_diag_vs_vert	1.00
6	return_diag_vs_vert	1.00	phase_tb_asymmetry_diff	.77
7	return_S1_net	0.88	all_smoothness	0.65
8	return_S0_net	0.81	bottom_mean_near_peak_dur	0.56
9	phase_contemp_lr_frac_diff	0.80	interval_cv	0.54
10	return_contemp_lr_frac	0.74	right_max_rate_of_change	0.50
k=4: Effect size ($\eta^{2}$)				
1	primary_S1_net	1.00	primary_S1_net	1.00
2	primary_S3_net	1.00	primary_S3_net	1.00
3	primary_lr_asymmetry	1.00	primary_lr_asymmetry	1.00
4	primary_tb_asymmetry	1.00	primary_tb_asymmetry	1.00
5	primary_diag_vs_vert	1.00	primary_diag_vs_vert	1.00
6	return_contemp_vert_frac	0.88	return_contemp_vert_frac	0.88
7	return_n_contemp	0.85	return_n_contemp	0.85
8	phase_tb_asymmetry_diff	0.77	phase_tb_asymmetry_diff	0.77
9	top_mean_near_peak_dur	0.77	top_mean_near_peak_dur	0.77
10	phase_contemp_vert_frac_diff	0.77	phase_contemp_vert_frac_diff	0.77

**Table A4:** Feature importance for Stairs. Effect size: Cohen’s *d* for k=2, $\eta^{2}$ for k>2.

Rank	KMeans		Agglomerative	
	Feature	Score	Feature	Score
k=2: Effect size (Cohen’s *d*)				
1	bottom_max_rate_of_change	12.48	bottom_max_rate_of_change	12.48
2	top_max_rate_of_change	10.35	top_max_rate_of_change	10.35
3	right_max_rate_of_change	10.28	right_max_rate_of_change	10.28
4	all_max_rate_of_change	8.77	all_max_rate_of_change	8.77
5	bottom_range	7.88	bottom_range	7.88
6	left_max_rate_of_change	7.44	left_max_rate_of_change	7.44
7	top_range	5.51	top_range	5.51
8	right_range	4.89	right_range	4.89
9	left_range	4.40	left_range	4.40
10	lr_asymmetry	3.10	lr_asymmetry	3.10
k=3: Effect size ($\eta^{2}$)				
1	return_S2_net	0.96	return_S2_net	0.96
2	bottom_max_rate_of_change	0.95	bottom_max_rate_of_change	0.95
3	right_max_rate_of_change	0.92	right_max_rate_of_change	0.92
4	top_max_rate_of_change	0.92	top_max_rate_of_change	0.92
5	top_range	0.91	top_range	0.91
6	bottom_range	0.90	bottom_range	0.90
7	return_S1_net	0.89	return_S1_net	0.89
8	all_max_rate_of_change	0.89	all_max_rate_of_change	0.89
9	left_max_rate_of_change	0.85	left_max_rate_of_change	0.85
10	return_S3_net	0.85	return_S3_net	0.85
k=4: Effect size ($\eta^{2}$)				
1	return_S3_net	1.00	return_S2_net	0.96
2	left_mean_near_peak_dur	0.99	bottom_max_rate_of_change	0.96
3	return_S2_net	0.97	right_max_rate_of_change	0.93
4	bottom_max_rate_of_change	0.95	all_smoothness	0.93
5	right_max_rate_of_change	0.93	top_max_rate_of_change	0.93
6	top_max_rate_of_change	0.92	top_range	0.91
7	return_S0_net	0.92	all_max_rate_of_change	0.90
8	top_range	0.91	bottom_range	0.90
9	bottom_range	0.90	return_S1_net	0.90
10	all_max_rate_of_change	0.90	left_max_rate_of_change	0.86

**Table A5:** Feature importance for NormWalk. Effect size: Cohen’s *d* for k=2, $\eta^{2}$ for k>2.

Rank	KMeans		Agglomerative	
	Feature	Score	Feature	Score
k=2: Effect size (Cohen’s *d*)				
1	all_max_rate_of_change	4.28	all_max_rate_of_change	7.98
2	return_n_contemp	2.80	right_range	5.23
3	right_max_rate_of_change	2.67	right_max_rate_of_change	4.93
4	return_S5_net	2.65	all_range	4.29
5	right_range	2.30	left_max_rate_of_change	3.09
6	primary_S5_net	2.29	left_range	2.65
7	all_range	2.24	return_lr_asymmetry	2.65
8	left_max_rate_of_change	2.16	primary_S0_net	2.65
9	primary_S4_net	2.00	primary_S1_net	2.65
10	return_lr_asymmetry	1.95	primary_S2_net	2.65
k=3: Effect size ($\eta^{2}$)				
1	primary_S0_net	1.00	primary_S0_net	1.00
2	primary_S1_net	1.00	primary_S1_net	1.00
3	primary_S2_net	1.00	primary_S2_net	1.00
4	primary_S3_net	1.00	primary_S3_net	1.00
5	primary_diag_vs_vert	1.00	primary_diag_vs_vert	1.00
6	return_S2_net	1.00	return_S2_net	1.00
7	return_S5_net	1.00	return_S5_net	1.00
8	bottom_range	0.98	bottom_range	0.98
9	right_range	0.95	right_range	0.95
10	return_S4_net	0.95	return_S4_net	0.95
k=4: Effect size ($\eta^{2}$)				
1	primary_S0_net	1.00	primary_S0_net	1.00
2	primary_S1_net	1.00	primary_S1_net	1.00
3	primary_S2_net	1.00	primary_S2_net	1.00
4	primary_S3_net	1.00	primary_S3_net	1.00
5	primary_diag_vs_vert	1.00	primary_diag_vs_vert	1.00
6	return_S2_net	1.00	return_S2_net	1.00
7	return_S5_net	1.00	return_S5_net	1.00
8	bottom_range	0.98	bottom_range	0.98
9	return_S4_net	0.97	return_S4_net	0.97
10	return_tb_asymmetry	0.96	return_tb_asymmetry	0.96

**Table A6:** Feature importance for TabletFloor. Effect size: Cohen’s *d* for k=2, $\eta^{2}$ for k>2.

Rank	KMeans		Agglomerative	
	Feature	Score	Feature	Score
k=2: Effect size (Cohen’s *d*)				
1	return_S5_net	10.17	return_S5_net	10.17
2	return_contemp_vert_frac	6.51	return_contemp_vert_frac	6.51
3	return_S1_net	5.84	return_S1_net	5.84
4	return_n_contemp	5.48	return_n_contemp	5.48
5	return_contemp_diag_frac	5.19	return_contemp_diag_frac	5.19
6	peak_amplitude_cv	2.74	peak_amplitude_cv	2.74
7	interval_cv	2.52	interval_cv	2.52
8	top_max_rate_of_change	2.49	top_max_rate_of_change	2.49
9	primary_S3_net	2.47	primary_S3_net	2.47
10	left_max_rate_of_change	2.46	left_max_rate_of_change	2.46
k=3: Effect size ($\eta^{2}$)				
1	return_contemp_diag_frac	0.96	return_S5_net	0.96
2	return_S5_net	0.95	return_contemp_diag_frac	0.94
3	return_contemp_vert_frac	0.91	return_n_contemp	0.88
4	return_contemp_lr_frac	0.89	return_contemp_vert_frac	0.88
5	return_S1_net	0.89	return_S1_net	0.85
6	return_n_contemp	0.89	return_S3_net	0.83
7	phase_contemp_lr_frac_diff	0.76	left_range	0.58
8	left_range	0.61	peak_amplitude_cv	0.57
9	return_S3_net	0.60	primary_S3_net	0.57
10	peak_amplitude_cv	0.57	lr_asymmetry	0.50
k=4: Effect size ($\eta^{2}$)				
1	top_max_rate_of_change	0.98	return_S3_net	1.00
2	all_range	0.97	return_S5_net	0.98
3	return_S5_net	0.96	top_max_rate_of_change	0.95
4	interval_cv	0.92	interval_cv	0.95
5	return_contemp_vert_frac	0.90	return_contemp_diag_frac	0.94
6	left_range	0.90	top_mean_near_peak_dur	0.91
7	return_contemp_diag_frac	0.89	return_S2_net	0.89
8	right_range	0.87	return_n_contemp	0.88
9	return_S1_net	0.87	return_contemp_vert_frac	0.88
10	return_n_contemp	0.84	return_S1_net	0.87

**Table A7:** Feature importance for TabletCabinet. Effect size: Cohen’s *d* for k=2, $\eta^{2}$ for k>2.

Rank	KMeans		Agglomerative	
	Feature	Score	Feature	Score
k=2: Effect size (Cohen’s *d*)				
1	return_contemp_vert_frac	24.84	return_contemp_vert_frac	24.84
2	phase_contemp_vert_frac_diff	17.58	phase_contemp_vert_frac_diff	17.58
3	right_mean_near_peak_dur	10.10	right_mean_near_peak_dur	10.10
4	phase_contemp_diag_frac_diff	9.57	phase_contemp_diag_frac_diff	9.57
5	all_mean_near_peak_dur	8.81	all_mean_near_peak_dur	8.81
6	primary_n_contemp	8.37	primary_n_contemp	8.37
7	return_contemp_diag_frac	7.66	return_contemp_diag_frac	7.66
8	left_mean_near_peak_dur	7.56	left_mean_near_peak_dur	7.56
9	return_n_contemp	5.95	return_n_contemp	5.95
10	peak_amplitude_cv	5.75	peak_amplitude_cv	5.75
k=3: Effect size ($\eta^{2}$)				
1	return_contemp_diag_frac	0.99	return_contemp_vert_frac	0.99
2	return_contemp_vert_frac	0.99	phase_contemp_vert_frac_diff	0.97
3	phase_contemp_vert_frac_diff	0.98	phase_contemp_diag_frac_diff	0.94
4	phase_contemp_diag_frac_diff	0.98	return_contemp_diag_frac	0.92
5	return_n_contemp	0.98	right_mean_near_peak_dur	0.92
6	left_mean_near_peak_dur	0.97	all_mean_near_peak_dur	0.91
7	phase_contemp_lr_frac_diff	0.94	left_mean_near_peak_dur	0.90
8	all_mean_near_peak_dur	0.94	primary_n_contemp	0.89
9	right_mean_near_peak_dur	0.93	return_n_contemp	0.85
10	return_contemp_lr_frac	0.93	peak_amplitude_cv	0.80
k=4: Effect size ($\eta^{2}$)				
1	return_contemp_vert_frac	0.99	primary_S5_net	1.00
2	phase_contemp_vert_frac_diff	0.97	return_contemp_diag_frac	0.99
3	right_mean_near_peak_dur	0.96	return_contemp_vert_frac	0.99
4	right_max_rate_of_change	0.95	phase_contemp_vert_frac_diff	0.98
5	all_mean_near_peak_dur	0.95	primary_diag_vs_vert	0.98
6	primary_S1_net	0.94	phase_contemp_diag_frac_diff	0.98
7	phase_contemp_diag_frac_diff	0.93	return_n_contemp	0.98
8	top_max_rate_of_change	0.91	left_mean_near_peak_dur	0.97
9	left_mean_near_peak_dur	0.91	phase_contemp_lr_frac_diff	0.94
10	primary_n_contemp	0.90	all_mean_near_peak_dur	0.94

**Table A8:** Top 10 discriminating features per movement by $\eta^{2}$ across subgroups A, B, and C.

**WeightedSuitcaseRight**					**WeightedSuitcaseLeft**				
**Feature**	$\eta^{2}$	**A**	**B**	**C**	**Feature**	$\eta^{2}$	**A**	**B**	**C**
Left range	0.88	3.53	2.70	1.37	Overall range	0.88	9.53	8.39	3.88
Left max. rate of chg.	0.82	6.46	6.11	2.83	Left range	0.84	3.14	1.84	0.96
Overall range	0.82	7.77	7.22	3.32	Overall max. rate of chg.	0.63	15.06	14.57	8.10
Ret. lower right (S6) net	0.76	−3.00	0.00	0.20	Right range	0.63	3.58	3.86	1.97
Prim. upper-left (S1) net	0.67	1.00	−0.33	0.00	Left max. rate of chg.	0.54	4.31	2.98	1.71
Overall max. rate of chg.	0.60	14.97	15.93	7.25	Right max. rate of chg.	0.51	6.50	6.87	4.11
LR asymmetry	0.48	0.95	0.43	0.20	Phase LR asym. diff.	0.48	0.00	1.00	1.11
Prim. TB asym.	0.48	1.00	−0.33	−0.40	LR asymmetry	0.30	−0.38	−1.06	−0.56
Ret. contemp. diag. frac.	0.45	0.36	0.22	0.41	Interval CV	0.30	0.13	0.15	0.26
Peak amplitude CV	0.44	0.05	0.26	0.06	Top near-peak dur.	0.29	0.65	0.43	0.30
**Stairs**					**NormWalk**				
**Feature**	$\eta^{2}$	**A**	**B**	**C**	**Feature**	$\eta^{2}$	**A**	**B**	**C**
Ret. upper-left (S1) net	0.72	−2.00	0.00	0.20	Top near-peak dur.	0.61	0.37	0.19	0.17
Overall range	0.60	4.62	6.67	2.51	Ret. lower right (S6) net	0.57	0.00	−0.67	0.00
LR asymmetry	0.57	0.05	−0.42	−0.07	Phase LR asym. diff.	0.54	0.00	0.03	0.11
Right range	0.47	1.53	3.18	1.10	Overall near-peak dur.	0.53	0.42	0.15	0.17
Overall max. rate of chg.	0.44	4.51	11.72	3.67	Ret. LR asym.	0.50	0.00	0.56	−0.40
Prim. mid-right net	0.43	0.00	−0.33	0.80	Overall max. rate of chg.	0.43	3.39	8.82	4.29
Ret. mid-left net	0.42	−2.00	1.33	−0.60	Prim. contemp. vert. frac.	0.42	0.46	0.39	0.53
Prim. upper-left (S1) net	0.40	1.50	0.00	−0.40	Overall range	0.42	3.30	5.08	1.72
Ret. mid-right net	0.40	2.50	−0.67	0.40	Prim. contemp. LR frac.	0.39	0.15	0.21	0.15
Right max. rate of chg.	0.39	1.58	4.86	1.56	Right range	0.32	1.30	2.39	0.89
**TabletFloor**					**TabletCabinet**				
**Feature**	$\eta^{2}$	**A**	**B**	**C**	**Feature**	$\eta^{2}$	**A**	**B**	**C**
Ret. lower right (S6) net	0.94	3.50	0.00	−0.20	Left range	0.83	5.54	4.06	1.95
Ret. contemp. vert. frac.	0.90	0.50	0.40	0.41	Overall range	0.79	13.27	15.49	5.82
Ret. contemp. diag. frac.	0.89	0.30	0.40	0.42	Overall max. rate of chg.	0.72	25.69	31.28	9.95
Left range	0.89	7.73	5.97	2.94	LR asymmetry	0.70	0.98	−1.17	−0.40
Overall range	0.88	14.85	20.80	9.00	Left max. rate of chg.	0.69	10.82	8.23	3.02
Right range	0.86	5.99	8.59	3.72	Ret. lower right (S6) net	0.55	−0.50	−0.67	0.80
Ret. upper-right net	0.86	−3.50	0.33	0.60	Right range	0.54	3.80	6.40	2.70
Ret. *n* contemp. links	0.84	10.00	14.33	14.20	Right max. rate of chg.	0.52	6.54	12.79	4.50
Overall max. rate of chg.	0.69	27.12	30.50	14.31	Prim. lower right (S6) net	0.44	−0.50	0.00	0.00
Top range	0.68	3.58	6.63	2.64	Top max. rate of chg.	0.43	3.85	9.08	3.76

## References

[1] Collaborators G.L.B.P. (2023). Global, regional, and national burden of low back pain, 1990–2020, its attributable risk factors, and projections to 2050: A systematic analysis of the Global Burden of Disease Study 2021. Lancet Rheumatol..

[2] Yang N., Di J., Wang W., Feng H. (2025). Global burden of low back pain from 1990 to 2021: A comprehensive analysis of risk factors and trends using the Global Burden of Disease Study 2021. BMC Public Health.

[3] Gombatto S.P., Bailey B., Bari M., Bouchekara J., Holmes A., Lenz S., Simmonds K., Vonarb A., Whelehon K., Batalla C.R. (2024). Identifying Clinical Phenotypes in People Who Are Hispanic/Latino with Chronic Low Back Pain: Use of Sensor-Based Measures of Posture and Movement, Pain, and Psychological Factors. Phys. Ther..

[4] Wernli K., Tan J.S., O’Sullivan P., Smith A., Campbell A., Kent P. (2020). Does Movement Change When Low Back Pain Changes? A Systematic Review. J. Orthop. Sport. Phys. Ther..

[5] Dankaerts W., O’Sullivan P., Burnett A., Straker L., Davey P., Gupta R. (2009). Discriminating Healthy Controls and Two Clinical Subgroups of Nonspecific Chronic Low Back Pain Patients Using Trunk Muscle Activation and Lumbosacral Kinematics of Postures and Movements: A Statistical Classification Model. Spine.

[6] Gombatto S.P., Collins D.R., Sahrmann S.A., Engsberg J.R., Van Dillen L.R. (2007). Patterns of Lumbar Region Movement During Trunk Lateral Bending in 2 Subgroups of People with Low Back Pain. Phys. Ther..

[7] Henchoz Y., Kai-Lik So A. (2008). Exercise and nonspecific low back pain: A literature review. Jt. Bone Spine.

[8] Nelson B.W., O’Reilly E., Miller M., Hogan M., Wegner J.A., Kelly C. (1995). The clinical effects of intensive, specific exercise on chronic low back pain: A controlled study of 895 consecutive patients with 1-year follow up. Orthopedics.

[9] Marich A.V., Lanier V.M., Salsich G.B., Lang C.E., Van Dillen L.R. (2018). Immediate Effects of a Single Session of Motor Skill Training on the Lumbar Movement Pattern During a Functional Activity in People with Low Back Pain: A Repeated-Measures Study. Phys. Ther..

[10] Burjawi T., El-Ansary D., Farragher J., Tirosh O., Pranata A. (2026). Machine learning accuracy for assessment of functional movement in Low back pain based on clinically applicable performance Metrics: A systematic review. Int. J. Med. Inform..

[11] Lin Y.A., Zhao Y., Wang L., Park Y., Yeh Y.J., Chiang W.H., Loh K.J. (2021). Graphene K-Tape Meshes for Densely Distributed Human Motion Monitoring. Adv. Mater. Technol..

[12] Wyckoff E., Gombatto S.P., Velazquez Y., Godino J., Patrick K., Farcas E., Loh K.J. (2025). Carbon Nanotube Elastic Fabric Motion Tape Sensors for Low Back Movement Characterization. Sensors.

[13] Spiegel S., Wyckoff E., Barolo J., Lee A., Farcas E., Godino J., Patrick K., Loh K.J., Gombatto S.P. (2024). Motion Tape Strain During Trunk Muscle Engagement in Young, Healthy Participants. Sensors.

[14] Runge J. (2022). Discovering contemporaneous and lagged causal relations in autocorrelated nonlinear time series datasets. arXiv.

[15] Ikotun A.M., Ezugwu A.E., Abualigah L., Abuhaija B., Heming J. (2023). K-means clustering algorithms: A comprehensive review, variants analysis, and advances in the era of big data. Inf. Sci..

[16] Murtagh F., Legendre P. (2014). Ward’s Hierarchical Agglomerative Clustering Method: Which Algorithms Implement Ward’s Criterion?. J. Classif..

[17] Balcan M.F., Liang Y., Gupta P. (2014). Robust Hierarchical Clustering. J. Mach. Learn. Res..

[18] Von Luxburg U. (2007). A tutorial on spectral clustering. Stat. Comput..

[19] Schaller A., Rudolf K., Dejonghe L., Grieben C., Froboese I. (2016). Influencing Factors on the Overestimation of Self-Reported Physical Activity: A Cross-Sectional Analysis of Low Back Pain Patients and Healthy Controls. BioMed Res. Int..

[20] Cuesta-Vargas A.I., Galán-Mercant A., Williams J.M. (2010). The use of inertial sensors system for human motion analysis. Phys. Ther. Rev..

[21] Kianifar R., Joukov V., Lee A., Raina S., Kulić D. (2019). Inertial measurement unit-based pose estimation: Analyzing and reducing sensitivity to sensor placement and body measures. J. Rehabil. Assist. Technol. Eng..

[22] García-de Villa S., Casillas-Pérez D., Jiménez-Martín A., García-Domínguez J.J. (2023). Inertial Sensors for Human Motion Analysis: A Comprehensive Review. IEEE Trans. Instrum. Meas..

[23] Hidalgo B., Nielens H., Gilliaux M., Hall T., Detrembleur C. (2014). Use of kinematic algorithms to distinguish people with chronic non-specific low back pain from asymptomatic subjects: A validation study. J. Rehabil. Med..

[24] Zheng X., Otten E., Reneman M.F., Lamoth C.J. (2025). Explaining deep learning models for age-related gait classification based on acceleration time series. Comput. Biol. Med..

[25] Shum G.L.K., Crosbie J., Lee R.Y.W. (2005). Symptomatic and Asymptomatic Movement Coordination of the Lumbar Spine and Hip During an Everyday Activity. Spine.

[26] Wade L., Needham L., McGuigan P., Bilzon J. (2022). Applications and limitations of current markerless motion capture methods for clinical gait biomechanics. PeerJ.

[27] Mazzone B., Wood R., Gombatto S. (2016). Spine Kinematics During Prone Extension in People with and Without Low Back Pain and Among Classification-Specific Low Back Pain Subgroups. J. Orthop. Sport. Phys. Ther..

[28] Gombatto S.P., D’Arpa N., Landerholm S., Mateo C., O’Connor R., Tokunaga J., Tuttle L.J. (2017). Differences in kinematics of the lumbar spine and lower extremities between people with and without low back pain during the down phase of a pick up task, an observational study. Musculoskelet. Sci. Pract..

[29] Hernandez A., Gross K., Gombatto S. (2017). Differences in lumbar spine and lower extremity kinematics during a step down functional task in people with and people without low back pain. Clin. Biomech..

[30] Hebert J.J., Koppenhaver S.L., Walker B.F. (2011). Subgrouping Patients with Low Back Pain: A Treatment-Based Approach to Classification. Sport. Health.

[31] Abdollahi M., Ashouri S., Abedi M., Azadeh-Fard N., Parnianpour M., Khalaf K., Rashedi E. (2020). Using a Motion Sensor to Categorize Nonspecific Low Back Pain Patients: A Machine Learning Approach. Sensors.

[32] Naumzik C., Kongsted A., Vach W., Feuerriegel S. (2024). Data-driven subgrouping of patient trajectories with chronic diseases: Evidence from low back pain. arXiv.

[33] Van Dillen L.R., Sahrmann S.A., Norton B.J., Caldwell C.A., McDonnell M.K., Bloom N.J. (2003). Movement System Impairment-Based Categories for Low Back Pain: Stage 1 Validation. J. Orthop. Sport. Phys. Ther..

[34] Han Y., Tao Q., Zhang X. (2025). Multijoint Continuous Motion Estimation for Human Lower Limb Based on Surface Electromyography. Sensors.

[35] Martins J., Cerqueira S.M., Catarino A.W., Silva A.F.d., Rocha A.M., Vale J., Ângelo M., Santos C.P. (2024). Integrating sEMG and IMU Sensors in an e-Textile Smart Vest for Forward Posture Monitoring: First Steps. Sensors.

[36] Ekstrom R.A., Osborn R.W., Hauer P.L. (2008). Surface Electromyographic Analysis of the Low Back Muscles During Rehabilitation Exercises. J. Orthop. Sport. Phys. Ther..

[37] Aarotale P.N., Rattani A. Machine Learning-based sEMG Signal Classification for Hand Gesture Recognition. Proceedings of the 2024 IEEE International Conference on Bioinformatics and Biomedicine (BIBM).

[38] Jiang N., Luk K.D.K., Hu Y. (2017). A Machine Learning-based Surface Electromyography Topography Evaluation for Prognostic Prediction of Functional Restoration Rehabilitation in Chronic Low Back Pain. Spine.

[39] Yun W.S., Kim H., Ahn J.H., Park Y.B., Park Y.J. (2015). Individual characteristics of reliable lumbar coupling motions. Eur. Spine J..

[40] Ricci L., Taffoni F., Formica D. (2016). On the Orientation Error of IMU: Investigating Static and Dynamic Accuracy Targeting Human Motion. PLoS ONE.

[41] Zhang Y., Haghighi P.D., Burstein F., Yap L.W., Cheng W., Yao L., Cicuttini F. (2020). Electronic Skin Wearable Sensors for Detecting Lumbar–Pelvic Movements. Sensors.

[42] Arvanitidis M., Jiménez-Grande D., Haouidji-Javaux N., Falla D., Martinez-Valdes E. (2022). People with chronic low back pain display spatial alterations in high-density surface EMG-torque oscillations. Sci. Rep..

[43] Lee A., Wyckoff E., Farcas E., Godino J., Patrick K., Spiegel S., Yu R., Kumar A., Loh K.J., Gombatto S. (2024). Preliminary Validity and Acceptability of Motion Tape for Measuring Low Back Movement: Mixed Methods Study. JMIR Rehabil. Assist. Technol..

[44] Loh K.J., Huang S.C., Lin Y.A. Enhancing Warfighter Marksmanship Performance using Motion Tape Elastic Fabric Sensors. Proceedings of the Interservice/Industry Training, Simulation, and Education Conference.

[45] Huang S.C., Lin Y.A., Pierce T., Wyckoff E., Loh K.J. (2023). Measuring the Golf Swing Pattern Using Motion Tape for Feedback and Fault Detection. Structural Health Monitoring 2023.

[46] Levy J., Lalwani A., Wyckoff E., Loh K.J., Gombatto S.P., Yu R., Farcas E. (2026). Assessing Low Back Movement with Motion Tape Sensor Data Through Deep Learning. Sensors.

[47] Deyo R.A., Dworkin S.F., Amtmann D., Andersson G., Borenstein D., Carragee E., Carrino J., Chou R., Cook K., Delitto A. (2015). Report of the NIH Task Force on Research Standards for Chronic Low Back Pain. Phys. Ther..

[48] Zhang X., Jia Y., Song M., Wang R. (2024). Similarity and Dissimilarity Guided Co-association Matrix Construction for Ensemble Clustering. arXiv.

[49] Siebers H.L., Alrawashdeh W., Betsch M., Migliorini F., Hildebrand F., Eschweiler J. (2021). Comparison of different symmetry indices for the quantification of dynamic joint angles. BMC Sport. Sci. Med. Rehabil..

[50] Błażkiewicz M., Wiszomirska I., Wit A. (2014). Comparison of four methods of calculating the symmetry of spatial-temporal parameters of gait. Acta Bioeng. Biomech..

[51] Chan L.C., Yan J., Zhang Y.C., Jiang T., Zhang A.Y., Li H.H.T., So B., Huang W., Zheng Y., Chan P.K. (2026). Smartphone-derived joint angular velocities in sit-to-stand motion provide a spatiotemporal marker for symptomatic knee osteoarthritis. Commun. Med..

[52] Spirtes P., Glymour C.N., Scheines R. (2000). Causation, Prediction, and Search.

[53] Miersch P., Günther W., Runge J., Zscheischler J. (2025). Evaluating the Robustness of PCMCI+ for Causal Discovery of Flood Drivers. Artif. Intell. Earth Syst..

[54] Silfwerbrand L., Koike Y., Nyström P., Gingnell M. (2024). Directed causal effect with PCMCI in hyperscanning EEG time series. Front. Neurosci..

[55] Krich C., Runge J., Miralles D.G., Migliavacca M., Perez-Priego O., El-Madany T., Carrara A., Mahecha M.D. (2020). Estimating causal networks in biosphere–atmosphere interaction with the PCMCI approach. Biogeosciences.

[56] Saggioro E., Wiljes J.d., Kretschmer M., Runge J. (2020). Reconstructing regime-dependent causal relationships from observational time series. Chaos Interdiscip. J. Nonlinear Sci..

[57] Mokhtari F., Akhlaghi M.I., Simpson S.L., Wu G., Laurienti P.J. (2019). Sliding window correlation analysis: Modulating window shape for dynamic brain connectivity in resting state. NeuroImage.

[58] Luxburg U.v. (2010). Clustering Stability: An Overview. Found. Trends^®^ Mach. Learn..

[59] Kearns M., Ron D. (1997). Algorithmic stability and sanity-check bounds for leave-one-out cross-validation. Proceedings of the Tenth Annual Conference on Computational Learning Theory.

[60] Liu T., Yu H., Blair R.H. (2022). Stability estimation for unsupervised clustering: A review. WIREs Comput. Stat..

[61] Berikov V., Pestunov I. (2017). Ensemble clustering based on weighted co-association matrices: Error bound and convergence properties. Pattern Recognit..

[62] Fred A.L.N., Jain A.K. (2005). Combining Multiple Clusterings Using Evidence Accumulation. IEEE Trans. Pattern Anal. Mach. Intell..

[63] Goswami M., Szafer K., Choudhry A., Cai Y., Li S., Dubrawski A. (2024). MOMENT: A Family of Open Time-series Foundation Models. arXiv.

[64] Ansari A.F., Stella L., Turkmen C., Zhang X., Mercado P., Shen H., Shchur O., Rangapuram S.S., Arango S.P., Kapoor S. (2024). Chronos: Learning the Language of Time Series. arXiv.

[65] Richardson J.T.E. (2011). Eta squared and partial eta squared as measures of effect size in educational research. Educ. Res. Rev..

[66] Tomašev N., Radovanović M., Celebi M.E., Aydin K. (2016). Clustering Evaluation in High-Dimensional Data. Unsupervised Learning Algorithms.

